# Mapping of anaemia prevalence among pregnant women in Kenya (2016–2019)

**DOI:** 10.1186/s12884-020-03380-2

**Published:** 2020-11-23

**Authors:** Julius Nyerere Odhiambo, Benn Sartorius

**Affiliations:** 1 Discipline of Public Health Medicine, College of Health Sciences, University of KwaZulu-Natal, Howard College Campus, 2nd Floor George Campbell Building, Durban, 4001 South Africa; 2 Department of Disease Control, Faculty of Infectious and Tropical Diseases, London School of Hygiene and Tropical Medicine, London, UK; 3 Department of Health Metrics Sciences, University of Washington, Seattle, USA

**Keywords:** Maternal anaemia, Sub-county, Conditional autoregressive model, Prevalence, Bayesian inference, Policy, Kenya

## Abstract

**Background:**

Reducing the burden of anaemia is a critical global health priority that could improve maternal outcomes amongst pregnant women and their neonates. As more counties in Kenya commit to universal health coverage, there is a growing need for optimal allocation of the limited resources to sustain the gains achieved with the devolution of healthcare services. This study aimed to describe the spatio-temporal patterns of maternal anaemia prevalence in Kenya from 2016 to 2019.

**Methods:**

Quarterly reported sub-county level maternal anaemia cases from January 2016 – December 2019 were obtained from the Kenyan District Health Information System. A Bayesian hierarchical negative binomial spatio-temporal conditional autoregressive (CAR) model was used to estimate maternal anaemia prevalence by sub-county and quarter. Spatial and temporal correlations were considered by assuming a conditional autoregressive and a first-order autoregressive process on sub-county and seasonal specific random effects, respectively.

**Results:**

The overall estimated number of pregnant women with anaemia increased by 90.1% (95% uncertainty interval [95% UI], 89.9–90.2) from 155,539 cases in 2016 to 295,642 cases 2019. Based on the WHO classification criteria, the proportion of sub-counties with normal prevalence decreased from 28.0% (95% UI, 25.4–30.7) in 2016 to 5.4% (95% UI, 4.1–6.7) in 2019, whereas moderate anaemia prevalence increased from 16.8% (95% UI, 14.7–19.1) in 2016 to 30.1% (95% UI, 27.5–32.8) in 2019 and severe anaemia prevalence increased from 7.0% (95% UI, 5.6–8.6) in 2016 to 16.6% (95% UI, 14.5–18.9) in 2019. Overall, 45.1% (95% UI: 45.0–45.2) of the estimated cases were in malaria-endemic sub-counties, with the coastal endemic zone having the highest proportion 72.8% (95% UI: 68.3–77.4) of sub-counties with severe prevalence.

**Conclusion:**

As the number of women of reproductive age continues to grow in Kenya, the use of routinely collected data for accurate mapping of poor maternal outcomes remains an integral component of a functional maternal health strategy. By unmasking the sub-county disparities often concealed by national and county estimates, our study findings reiterate the importance of maternal anaemia prevalence as a metric for estimating malaria burden and offers compelling policy implications for achieving national nutritional targets.

**Supplementary Information:**

The online version contains supplementary material available at 10.1186/s12884-020-03380-2.

## Background

Maternal anaemia, defined as the haemoglobin concentration below 11 g per decilitre (g/dL) [1]; is a persistent global health concern, a leading cause of disability in pregnant women and remains a major risk factor for adverse pregnancy outcomes [2–4]. In 2015, the World Health Organization (WHO) estimated 273 million children and 529 million women to be affected worldwide [5], accounting for approximately 8.8% of global disability-adjusted life years [6]. While anaemia affects women globally, the major burden of maternal anaemia has remained unacceptably high in Low and Middle-Income Countries (LMICs) [7], with countries in Sub-Saharan Africa (SSA) and South East Asia disproportionately affected [8]. In its 2016 Global Nutrition Report, the WHO target of a 50% reduction of maternal anaemia by 2025 was reported to be 100 years behind schedule [9, 10] despite the considerable economic and scientific advancement over the past two decades. This ambitious, yet achievable, target still calls for a renewed focus on the optimal approaches necessary to improve the quality of care provided to women and their infants.

In pregnancy, anaemia’s aetiology is complex and is aggravated by a host of factors involving the complex interaction of infectious disease [11], nutrition and inherited disorders [6, 12]. Previous reviews have associated maternal anaemia with an increased risk of adverse outcomes such as; low birth weight [13], preterm birth [14] as well as an increased risk of maternal and perinatal mortality [5]. In Kenya, maternal anaemia etiological diversity is affected by limited resources, leading to preventable morbidity and mortality at the sub-county level [15]. To address this burden, national policy guidelines and interventions on combined iron and folic acid supplementation for pregnant women have sought to improve both neonatal and maternal outcomes. This has been implemented through the goal-oriented and women-centred focused Antenatal Care (FANC) program, that recommends at least four scheduled comprehensive antenatal visits to promote the health of pregnant women and their infants. Additionally, FANC offers targeted assessments useful for identifying potential birth complications, treating established disease and availing information critical for a positive pregnancy experience [16, 17].

To orient the implementation of intervention initiatives, comparable sub-county (policy meaningful) estimates of maternal anaemia prevalence in Kenya would be useful. However, national and sub-national estimates in diverse epidemiological settings, ethnicities and socioeconomic strata is yet to be fully assessed and quantified using routinely collected data. This may due to data sparsity and the disproportionate sub-optimal coverage and adherence rates [18]. Nonetheless, Bayesian model-based predictions with the spatial and temporal covariates can be used to obtain reliable and stable sub-county estimates for maternal anaemia metrics [19, 20]. In the era of diminishing resources, understanding maternal anaemia trends at the sub-county level—at which services are planned, organised, and delivered— will assist local policymakers in deploying tailored, equity-oriented, and availing nutrition-specific interventions to women in high-risk areas. This will enable the country to monitor progress towards attainment of both national and global targets i.e. reducing by 50% the prevalence of anaemia among women of reproductive age by 2025 [3].

## Methods

### Study area

**Fig. 1:**
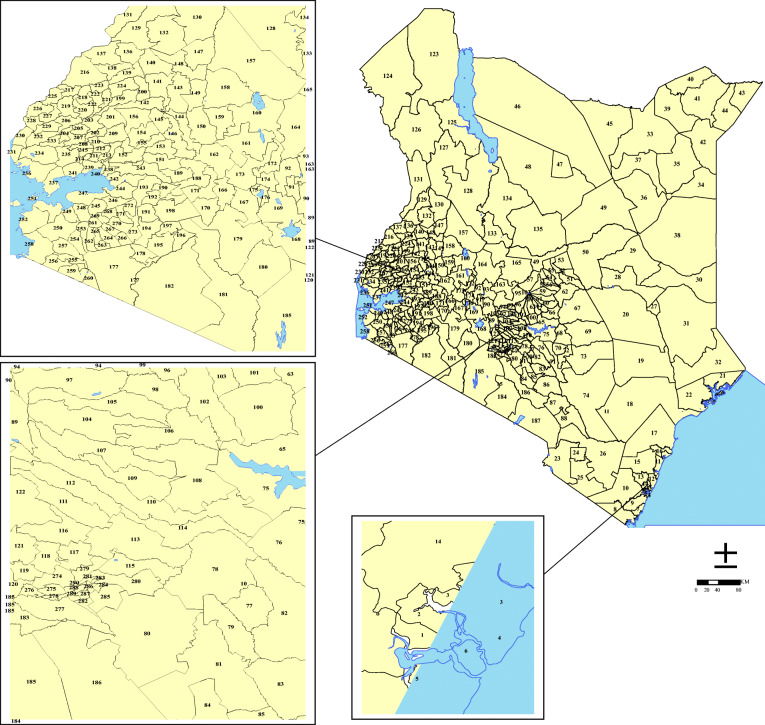
The map of Kenya showing 290 sub-counties (numbered), with the extents of major lakes and the Indian ocean shown in light blue. The names of counties, sub-counties and their malaria endemicity status are presented in Additional file 2. (Source: https://data.humdata.org/dataset/ken-administrative-boundaries)

Kenya covers an approximate area of 580,367 sq.km, with an estimated population of 47.9 million in 2018 [21]. Kenya has a predominantly agricultural economy with an emerging industrial base. It was ranked 146 out of 188 countries on the UN Human Development Index, based on life expectancy, adult literacy and per capita income in 2015 [22]. Kenya has a decentralized system of governance comprised of 47 semi-autonomous counties and 290 sub-counties (Fig. 1). The county is the most important administrative unit tasked with the provision of health services. It has three types of climatic zones namely: hot and wet covering areas along the Indian Ocean coastline, temperate covers areas toward the west and south-west of the country and the hot and dry climate covering the north and eastern parts of the country.

Kenya’s healthcare system is hierarchically structured across six levels of care with the community unit being the basic level [23]. This is followed by levels 2 and 3 - primary care services - which provide preventive and curative care, including health services for childbirth. These are followed by county referral health services (level 4 and 5) in specific counties and the national referral health services (level 6) focusing on curative and rehabilitation being at the peak [24].

### Healthcare utilization

Over the past decade; periodic population-based surveys have availed retrospective data important for planning and orienting policies. However, population-based surveys are capital intensive and might not be able to fully capture changes in today’s dynamic healthcare environment. In contrast, routinely collected data potentially avails real-time actionable data; critical for the design and implementation of optimal control and intervention efforts. Sustained improvements in both the national and sub-national reporting completeness [25, 26] over the first 5 years of routine District Health Information Software System version 2(DHIS2) implementation have previously been reported in Kenya. This is based on the percentage of facilities reporting any data consecutively at 3,6,9, or 12 months [27]. During 2012/13–2015/16 period, between 2016 and 2019, ANC coverage rates based on the DHIS2 ranged from 95 to 99%, with the coverage of first ANC visit reported being nearly universal [25].

### Data sources and assembly

In Kenya, routine facility-based data reported monthly into an online District Health Information System database, form the primary data source for national and sub-national planning, surveillance and program monitoring and evaluation [27]. Data on pregnant women presenting at public health facilities with anaemia (clinically diagnosed with Hb < 11 g/dL) between January 2016 and December 2019, from 290 sub-counties were extracted from the DHIS2 platform (https://hiskenya.org). Data prior to 2016 were excluded due to a major change incorporated into the harmonized (DHIS2) reporting system and subsequently adopted by the Ministry of Health (MOH) in 2016 [28].

Quarterly data were stratified into five malaria endemic zones and subsequently cleaned by checking for duplicates and other inconsistencies in both Excel 2013 (Microsoft Corporation, Seattle, WA) and Stata version 15 (Stata Corp LLC, College Station, TX). All the datasets were reconciled to 290 sub-county boundaries obtained from the humanitarian data exchange platform (https://data.humdata.org). To minimize the bias caused by facility utilisation rate at the sub-county level, the denominator was the number of new clients presenting at the health facility [29]. The analyses adheres to guidelines stipulated for accurate and transparent health estimates reporting (GATHER) [30] (Additional file 3).

### Bayesian spatio-temporal modelling

As the observed number of anaemia cases could be seasonal, a hierarchical negative binomial regression model with 16 quarters as time units, was used to explore the spatial and temporal dynamics of maternal anaemia in Kenya. The study adopted a model without fixed covariates as the initial step towards exploring the maternal anaemia risk distribution at the sub-county level.

Let *Hb*_*it*_ denote the number of observed/reported Hb < 11 g/dl cases at time *t*, where *i* = 1…290 (total number of sub-counties in Kenya) and *t* = 1, …, 16, (quarters between January 2016 – December 2019). Then conditional on the relative risk *π*_*it*_, *Hb*_*it*_ is assumed to be a product of independent negative binomial distributions with parameters *E*_*it*_* and r* (Eq.1). Here *E*_*it*_ relates to the expected number of cases in sub-county *i* at time *t*, and ***r*** is the overdispersion parameter. *Hb*_*it*_ approaches a Poisson distribution as ***r*** approaches 0. That is,

1 $$ {Hb}_{it}\mid {\pi}_{it}\sim \mathrm{NegBin}\left({E}_{it}{\pi}_{it},r\right) $$

The relative risk (*π*_*it*_) of maternal anaemia is then specified as a function of spatial random effects, temporal effects and spatio – temporal interaction effects [31].

$$ Log\left({\upmu}_{it}\right)= Log\left({E}_{it}\right)+ Log\left({\pi}_{it}\right) $$

Where *Log*(*E*_*it*_) is the offset and *Log*(*π*_*it*_) is modelled as;

$$ Log\left({\pi}_{it}\right)=\propto +{\lambda}_i+{\xi}_t+{v}_{it},i=1,\dots, 290;t=1,\dots, 16 $$

2 $$ {\pi}_{it}=\mathit{\exp}\left(\propto +{\lambda}_i+{\xi}_t+{v}_{it}\right) $$

Where ∝ is the global risk, *λ*_*i*_ is the main spatial effects, *ξ*_*t*_ is the main temporal effects, and the space-time interaction term is represented by *v*_*it*_. The random effects (*λ*_*i*_, *ξ*_*t*_) were assigned prior distributions across the space-time cube to better capture the underlying structure of maternal anaemia prevalence (Eq.2).

Assuming a Besag – York – Mollie (BYM) specification, the spatial dependency was formalised using an intrinsic conditional autoregressive structured model (ICAR) [32–34]. The Gamma, flat and normal prior distributions were used for precision parameters, intercept, and model coefficients respectively (Additional file 1). A two-chain Markov chain Monte Carlo simulation (MCMC) model with 71,000 iterations and a burn in of 4000 samples was implemented in the Bayesian software package WINBUGS [35] (available at http://www.mrc-bsu.cam.ac.uk/bugs/welcome.shtml. Maps of estimated prevalence rates were created in ArcMap 10.6.1 (ESRI Inc., Redlands, CA, USA).

### Model diagnostics

A random sample of 500 observed data points was drawn from the space-time cube was used to validate the predictive power of the model. The data with the removed points was re-inputted into WinBUGS and the posterior distribution of the predicted data points and observed values were compared. The correlation coefficient and scatterplots were then used to quantify the association of the predicted prevalence with the crude observed prevalence at sub-county level (Additional file 1, Fig. 1). Convergence of the model chains was assessed visually by inspecting the series plot of each parameter and also by using the Gelman-Rubin statistics [36]. (Additional file 1, Fig. 2).

**Fig. 2:**
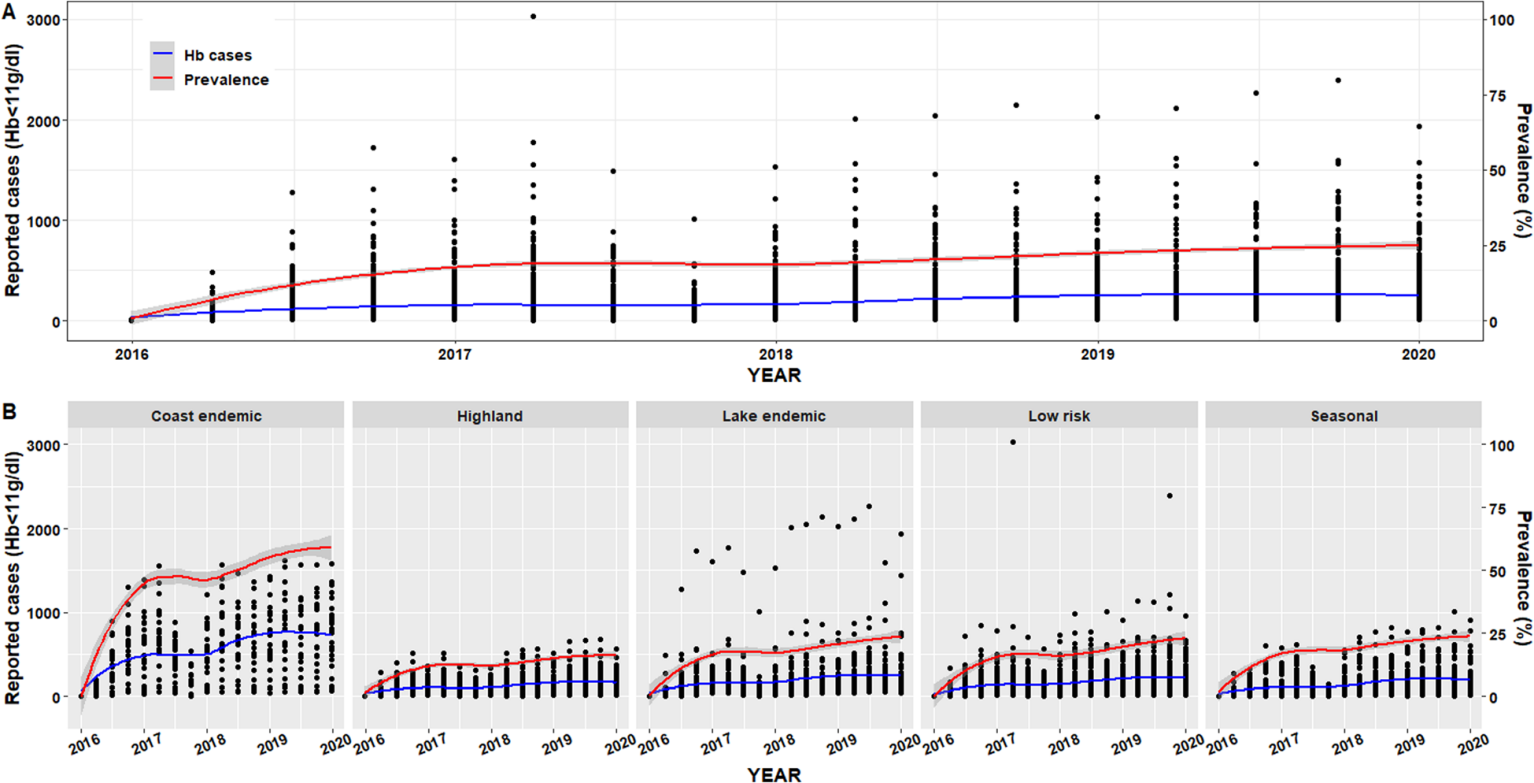
Posterior estimates of Hb cases and prevalence stratified by malaria endemicity. **a**: The blue and red line for estimated cases and prevalence respectively represent the lines of best fit according to the locally weighted scatterplot smoothing (loess). Shading indicates 95% UIs for estimated cases and prevalence. **b**: Posterior estimates of reported cases and median prevalence stratified by malaria endemicity between 2016 and 2019. (Source: author generated map)

## Results

### The burden of maternal anaemia in Kenya from 2016 to 2019

Overall, a total of 886,168 maternal anaemia cases were reported in 290 sub-county facilities from 2016 to 2019. Compared to 2016 estimates, the number of cases increased by 10.4% (95% UI: 10.2–10.5) to 171,682 cases in 2017, 69.3% (95% UI: 69.1–69.5) to 263,305 cases in 2018 and 90.1% (95% UI: 89.9–90.2) to 295,642 cases in 2019. The fourth quarter ranging from October to December which coincides with short rainfall season had the highest number of cases, with 235,295 (26.6%, (95% UI: 26.5–26.6)) cases recorded. This was followed by the second quarter ranging from April to June coinciding with the long rainfall season with 222,250 (25.1% (95% UI: 25.0–25.2) cases. The third quarter ranging from July to September had 215,273 (24.3% (95% UI: 24.2–24.4)) cases and first quarter ranging from January to March with 213,350 (24.1% (95% UI: 24.0–24.2)) cases, respectively.

The crude prevalence was 15.0% (95% UI: 14.9–15.1) in 2016 and increased over time by 20.5% (95% UI: 20.4–20.6) in 2017, 23.1% (95% UI: 23.0–23.2) in 2018 and 26.2% (26.1–26.3) in 2019.

In 2016, prevalence estimates ranged from 4.2% (95% UI: 4.1–4.3) in the first quarter to 23.0% (95% UI: 22.8–23.2) in the fourth quarter (Fig. 4). The proportion of sub-counties with normal prevalence was 28.0% (95% UI: 25.4–30.7), mild prevalence was 48.2% (95% UI: 45.3–51.1), moderate prevalence was 16.8% (95% UI: 14.7–19.1) severe prevalence was 7.0% (95% UI: 5.6–8.6 (Table 1).

**Table 1:** Proportion of sub-counties categorized by public health significance of anaemia: Prevalence below 5% was considered to be normal, prevalence between 5 and 19.9% was a mild public health problem, prevalence between 20 and 39.9% was moderate public health problem whereas prevalence ≥40% was considered a severe public health problem

Public Health Problem^**a**^	Year			
	2016	2017	2018	2019
**Normal (< 5.0%)**	28.0 (25.4–30.7)	12.0 (10.1–13.9)	8.5 (6.9–10.1)	5.4 (4.1–6.7)
**Mild (5.0–19.9%)**	48.2 (45.3–51.1)	56.7 (53.9–59.6)	53.4 (50.5–56.2)	47.8 (45.0–50.8)
**Moderate (20.0–39.9%)**	16.8 (14.7–19.1)	20.8 (18.5–23.1)	24.0 (21.5–26.4)	30.1 (27.5–32.8)
**Severe (> 40.0%)**	7.0 (5.6–8.6)	10.5 (8.8–12.3)	14.2 (12.3–16.4)	16.6 (14.5–18.9)

^a^WHO recommendations for prevalence thresholds to define severity of anaemia

In 2017, maternal anaemia prevalence estimates ranged from 17.0% (95% UI: 16.8–17.2) in the third quarter to 21.7% (95% UI: 21.5–21.8) in the first quarter. Sub-counties with severe prevalence increased to 10.5% (95% UI: 8.8–12.3), whereas moderate prevalence increased to 20.8% (95% UI: 18.5–23.1) (Additional file 2, Table 2). Compared to the 2016 estimates, the proportion of sub-counties with normal prevalence appears to have decreased by 86.1% (95% UI: 81.4–90.7) and by 38.5% (95% UI: 25.2–51.7) in the first and second quarter respectively (Fig. 4).

**Table 2:** Proportion of maternal anaemia prevalence stratified by malaria endemic zones, 2016–2019: Coast and Lake endemic sub-counties have stable malaria transmission throughout the year. Highland epidemic has seasonal transmission patterns with considerable year-to-year variation whereas Low risk sub-counties have low temperatures unsuitable for the malaria parasite sporogonic cycle. Seasonal transmission zone has short periods of intense malaria transmission during the rainfall seasons

Endemicity	Public Health Significance (95% UI)			
	Normal (< 5.0%)	Mild (5.0–19.9%)	Moderate (20.0–39.9%)	Severe (> 40.0%)
**Coast**	1.1% (0.0–2.2)	9.0% (6.1–11.9)	17.1% (13.3–21.0)	72.8% (68.3–77.4)
**Highland**	20.1% (17.9–23.1)	58.1% (54.9–61.3)	20.2% (17.6–22.8)	1.2% (0.1–1.9)
**Lake**	8.8% (10.1–14.1)	55.8% (52.7–58.8)	22.5% (19.9–25.1)	9.7% (7.8–11.5)
**Low risk**	12.6% (10.8–14.4)	58.2% (55.4–60.9)	23.1% (20.8–25.4)	6.2% (4.8–7.5)
**Seasonal**	13.5% (11.5–15.5)	48.8% (45.9–51.8)	27.3% (24.6–29.9)	10.4% (8.6–12.2)

In 2018, maternal anaemia prevalence estimates ranged from 22.2% (95% UI: 22.0–22.3) in the first quarter to 24.3% (95% UI: 24.1–24.5) in the fourth quarter (Fig. 4). The proportion of sub-counties with normal prevalence and mild prevalence thresholds decreased to 8.5% (95% UI: 6.9–10.1) and 53.4% (95% UI: 50.5–56.2) respectively. On the other hand, moderate and severe prevalence increased to 24.0% (95% UI: 21.5–26.4) and 14.2% (95% UI: 12.3–16.4) respectively (Table 1).

By 2019, the estimated maternal anaemia prevalence ranged from 24.6% (95% UI: 24.4–24.7) in the first quarter to 27.6% (95% UI: 27.5–27.8) in the third quarter (Fig. 4). Compared to the 2018 estimates, sub-counties with normal and mild prevalence decreased by 35.7% (95% UI: 26.2–45.2) and 10.3% (95% UI: 7.9–12.7) respectively. On the other hand, sub-counties with moderate and severe prevalence increased by 25.5% (95% UI: 20.4–30.7) and 17.0% (95% UI: 11.2–22.7).

### Spatial distribution of anaemia case counts and prevalence

**Fig. 3:**
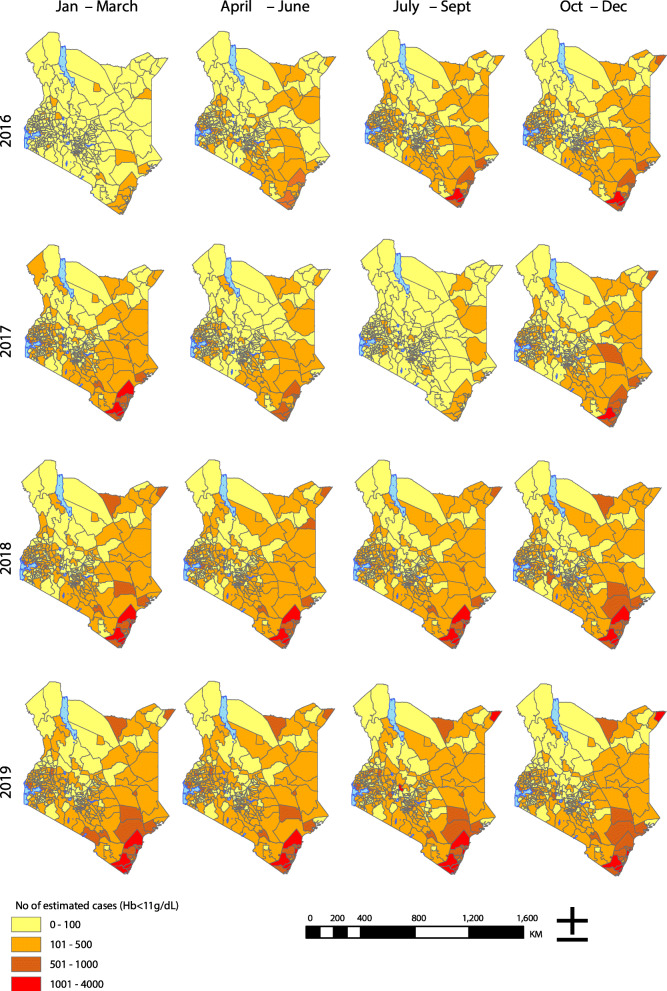
The spatial distribution of predicted Hb cases by subcounty in Kenya, 2016–2019 classified into four classes of 0–100 (light yellow), 101–500 (orange), 501–1000 (brown) and 1001–4000 (red). (Source: author generated map)

Disparities in anaemia prevalence and case counts were apparent in many sub-counties, reflecting the slow progress and the need for timely interventions. Overall, Kisumu Central sub-county had the highest proportion of estimated case counts with 3.1% (2.9–3.2), followed by Kinango 2.2% (2.0–2.3)), Matuga 1.9% (1.8–2.1), Likoni 1.8% (1.6–1.9), Magarini 1.6% (1.5–1.8). On the other hand, Ol Jorok, Lari, Turkana East, Turkana North and Narok East sub-counties had the least number of estimated cases over the study period (Fig. 3).

**Fig. 4:**
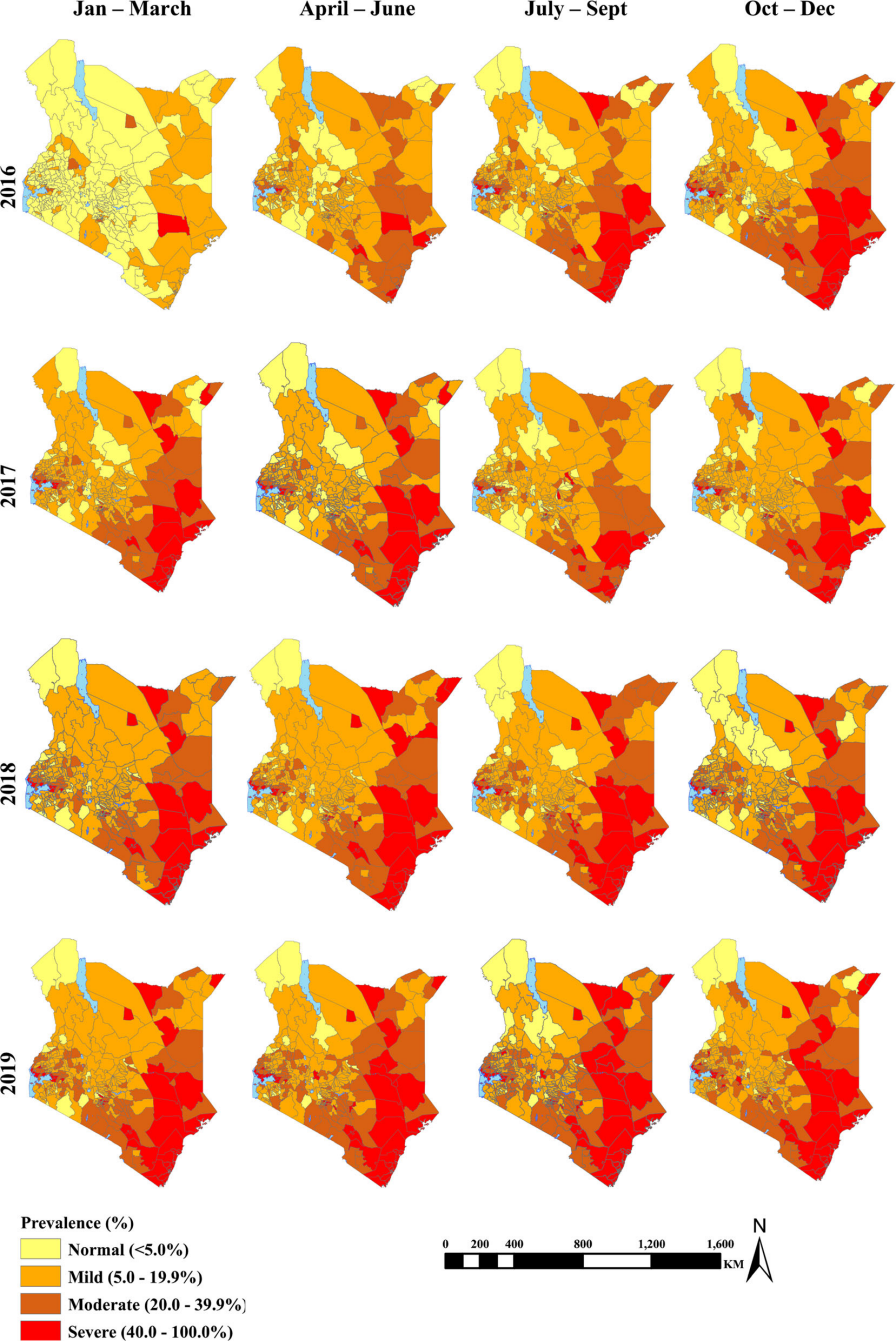
Map showing the estimated median prevalence of maternal anaemia in Kenya (2016–2019) using the Bayesian spatio-temporal CAR model. The classified into four classes based on WHO recommendations for defining anaemia prevalence thresholds. Below 5% (light yellow), 5.0–19.9% (orange), 20.0–39.9% (brown) and ≥ 40% (red). (Source: author generated map)

A continuous geographic disparity in maternal anaemia prevalence was evident from 2016 to 2019, with elevated prevalence exhibited in arid and semi-arid (ASA) sub-counties located along the Indian ocean coastline, Lake Victoria region and the North Eastern and Eastern regions. Elevated prevalence was dominated by sub-counties in Kwale, Kilifi, Mombasa, Lamu and Taita-Taveta counties that were located along the Indian ocean coastline. Highly populated sub-counties around Lake Victoria basin with elevated prevalence were Kisumu Central, Nyando, Seme and Nyakach, Kisumu West, Kisumu East, Bondo, Ugunja, Rarieda and Budalangi. In arid and semi-arid lands, elevated prevalence was observed in Fafi, Balambala, Lagdera, Dujis, Isiolo South, Kitui Central, Lafey, Mandera East, Moyale, Saku, Bura, Galole, Garsen, Wajir West, Wajir East, Wajir North, Kibwezi East, Kathiani and Kibwezi West sub-counties. Elevated prevalence was also observed in highly populated sub-counties located in Kiambu and Nairobi counties. These sub-counties were Kiambu Town, Ruiru, Kamukunji, Embakasi East, Embakasi South, Embakasi Central, and Kasarani. (Fig. 4).

### Maternal anaemia prevalence and malaria endemicity

The spatial distribution of maternal anaemia prevalence by malaria endemicity showed substantial heterogeneity in trends. Overall, 45.1% (95% UI: 45.0–45.2) of the estimated cases were reported in malaria-endemic sub-counties (Coast endemic and Lake endemic). This was followed by low risk sub-counties with 23.8% (95% UI: 23.7–23.9) of the cases, seasonal endemic sub-counties with 18.0% (95% UI: 17.9–18.1) of the cases and highland endemic sub-counties with 13.1% (95% UI: 13.0–13.2) of the cases. Coast endemic zone had the highest proportion 72.8% (95% UI: 68.3–77.4) of sub-counties with elevated prevalence followed by the seasonal endemic zones, lake endemic, low risk and highland endemic zones, respectively. (Table 2). In the Highland endemic zone, severe prevalence was dominated by Ainamoi sub-county in Kericho County.

## Discussion

We examined the disparities in maternal anaemia prevalence across sub-counties in Kenya from 2016 to 2019. Our estimates show a distinct pattern of elevated risk in arid and semi-arid sub-counties located in the North Eastern and Eastern parts, along the Indian ocean coastline, and Lake Victoria region over the study period. However, the diverse and increasing trends of maternal anaemia may be attributed to a range factors such as communicable and non-communicable diseases, regional dietary preferences, health care access and socio-economic factors. Thus, caution should be taken when interpreting the study results. To our knowledge, this study is the first to estimate maternal anaemia comprehensively at a fine geospatial scale in Kenya, and will bevital to the design and targeting of local-level maternal healthcare interventions.

### Concomitant contributors to maternal anaemia

The importance of maternal anaemia as a direct and indirect consequence of malaria and its prevalence among the vulnerable pregnant women is yet to be reported consistently as a metric of malaria transmission and burden in Kenya [37]. Similar to studies done in Sudan, Rwanda and Uganda [38–40], our study spatial trends suggests an association between malaria with maternal anaemia. Malaria-endemic sub-counties located along the Indian Ocean coastline and Lake Victoria region and seasonal endemic regions in the North Eastern and Eastern parts of Kenya dominated the proportion of sub-counties with elevated prevalence of maternal anaemia (Fig. 3). Evidence indicates that malaria control in endemic counties can improve mean haemoglobin levels in children and pregnant women, reducing the burden of severe anaemia by up to 60% [41, 42]. In the era of limited resources, integrated efforts addressing both malaria and anaemia in affected sub-counties would be an ideal avenue of improving the overall population health outcomes [43].

Human immunodeficiency virus (HIV) infection also exacerbates anaemia in pregnancy by compromising the mother’s immune system [42, 44] thereby exposing the pregnant mother to frequent and severe anaemia. By depleting the CD4+ T cells, HIV influences the rate of maternal anaemia progression [45, 46]. This is corroborated by a cohort study done in western Kenya (Lake endemic zone) associating malaria and HIV coinfection to doubling the risk of moderate-severe anaemia in pregnant women [47] suggesting a possible relationship. Anaemia is also considered a useful indicator of neglected disease burden and control [48]. Neglected tropical diseases (NTDs) such as hookworm infection [49], schistosomiasis [50] causes anaemia either directly through blood loss or indirectly through bone marrow suppression, haemolysis, inflammation [51, 52] also posing a devasting health burden to pregnant women. An estimated 40% of households in rural areas in Kenya rely on low-quality sources of drinking water such as unprotected wells, surface water and tanker trucks [53]. These sources are not only prone to pollution, but also provide conducive breeding grounds for causative agents of NTDs, which may ultimately contribute to maternal anaemia.

#### Micronutrient deficiencies

Nutritional induced anaemia results from the insufficient bioavailability of haemopoietic nutrients critical for the haemoglobin and erythrocyte synthesis [12, 54]. This is intense in pregnancy due to the additional nutritional demands associated with fetal growth [12]. The high demand of nutrients during pregnancy has bolstered global and national efforts towards micro-nutrient programming, supplementation and fortification initiatives. However, low ANC attendance and compliance with Iron Folic Acid supplementation (IFAS) has been reported in Kenya [55–58]. Pregnant women in pastoralists dominated sub-counties in the North and North Eastern parts of Kenya have low dietary diversity due to their high milk and meat consumption, that is compounded by irregular rainfall patterns stagnating agricultural production and inefficient food systems [59, 60].

#### Social, economic and cultural factors

Kenya’s growing population inhabits diverse sub-counties with different socio-economic development levels, health care needs and health-seeking behaviour [61]. Majority of the marginalised population live in the arid and semi-arid parts of Kenya, which tend to have a low density of healthcare facilities [62]. Women in these rural areas have a limited ability in seeking care, which might impact on their knowledge levels on the usefulness of critical maternal interventions. Consistent with findings from a multilevel study in Ethiopia [63], elevated prevalence of maternal anaemia mirrors the inequities between sub-counties, and this is starkly illustrated by sub-counties in the arid and semi-arid north of Kenya, areas around Lake Victoria, the rural north rift, and coastal region [64]. Interestingly, highly populated sub-counties in Nairobi and Kiambu characterised by low social-economic status/urban informal settlements also had severe prevalence reported over the study period.

### Limitations

Maternal anaemia clinical presentation is complex, and its risk mapping thereof without the covariates may compromise the accuracy of the map. Haemoglobin (Hb) measurements done during different semesters (first – fourth), parity, obstetrical complications and haematological disorders could have also impacted on the overall trend. The accuracy of our estimates was also dependent on the quality and extent of the data continuously obtained from health facilities and entered into the DHIS2 database. Given the aggregated nature of our input data, it was not feasible to fully explore the role of systemic problems affiliated with data capture, nor the extent to which causal inference can be made, due to the inability to distinguish between missing values (no data reported) and zero values (no events captured) within the study confines.

Underreporting of cases outside the formal health facility may have been missed, especially in sub-counties with high-prevalence due to overstretched resources, and this may have biased our analysis. Additionally, the Kenyan health system faced industrial action of multiple cadres involving doctors, nurses, and clinical officers in 2017 [65, 66]. This might have affected health care provision in the public health facilities leading to underreporting and consequently the underestimation of maternal anaemia true population prevalence. Thus, extra caution should be taken when interpreting the true maternal anaemia prevalence in Kenya. Additional research is needed to assess the dynamic interplay between nutrition, infectious disease, behavioural tendencies and social-economic factors is areas with elevated prevalence.

## Conclusion

Despite these limitations, our study supports the growing evidence base for precision public health data, based on routine health surveillance data and reiterate the importance of timely maternal anaemia prevalence estimate as a metric in malaria control. Sub-county estimates can be used to empower counties to benchmark on the gains in maternal health against other sub-counties as well as employ best practices advocated for by their peers. The elevated risk of maternal anaemia in malaria endemic sub-counties also calls into question the effectiveness of nationally initiated IFAS and other ANC programs intended to improve maternal health outcomes. Findings also provide a rationale for localised initiatives to complement global and national initiatives so as to meet the Global Nutrition Targets (GNTs) by 2025. Most importantly, in the era of data sparseness; the study provides a platform for triangulating routinely collected data with periodic survey-based estimates, so as to structure policy and steer precision public-health initiatives in a complete and unbiased manner.

## Supplementary Information

**Additional file 1.** Spatio-temporal modelling details.

**Additional file 2.** Additional data descriptions, methodological information and results.

**Additional file 3.** GATHER checklist.

## Data Availability

The datasets analysed during the current study are available in the Kenya District Health Information Software online database (DHIS2) repository, http://www.hiskenya.org. The shapefile used in the study is available in https://data.humdata.org/dataset/ken-administrative-boundaries.

## Abbreviations

ANC: Antenatal clinic
DHIS2: District Health Information Software System version 2
MCMC: Markov Chain Monte Carlo
MOH: Ministry of Health
IFAS: Iron Folic Acid supplementation
NTDs: Neglected tropical diseases
GDP: Gross Domestic Product
WHO: World Health Organization
ART: Antiretroviral therapy
CD4: Cluster of differentiation 4
