# Supplementary material for: Mapping of anaemia prevalence among pregnant women in Kenya (2016–2019)
Source: BMC Pregnancy Childbirth. 2020 Nov 23;20:711. doi: 10.1186/s12884-020-03380-2 (PMC7685542; doi:10.1186/s12884-020-03380-2)
Supplement: Supplementary file 1 — Additional file 1. Spatio-temporal modelling details. [file 12884_2020_3380_MOESM1_ESM.docx]

**Bayesian space-time modelling**

***Hierarchical spatio-temporal model structure***

The spatio-temporal model structure consists of three levels namely; the data level, process level and the parameter levels. The data level exhibits an independent conditional probability distribution subject to the process and modelling parameters whereas the process level determines the changes within the data level subject to the parameters.

***Data model***

A hierarchical negative binomial regression model was used to explore the spatial and temporal dynamics of maternal anaemia in Kenya.

Let $\boldsymbol{Hb}_{\boldsymbol{it}}$denotes the number of observed/reported Hb < 11g/dl cases at time $t$, where $i=1\ldots290$(total number of sub-counties in Kenya) and $t=1, \ldots, 16$, (quarters from 2016 – 2019). Then conditional on the relative risk $\pi_{it},$the data level is assumed to be a product of independent negative binomial distributions with parameters $\boldsymbol{E}_{\boldsymbol{it}}\boldsymbol{and r}$**.** Here $\boldsymbol{E}_{\boldsymbol{it}}$ relates to the expected number of cases in sub-county $i$ at time $t$, and $\boldsymbol{r}$ is the overdispersion parameter. $\boldsymbol{Hb}_{\boldsymbol{it}}$ approaches a Poisson distribution as $\boldsymbol{r}$ approaches 0. That is,

$\boldsymbol{Hb}_{\boldsymbol{it}}|\pi_{it}\sim NegBin(\boldsymbol{E}_{\boldsymbol{it}}\boldsymbol{\pi}_{it}\boldsymbol{,r)}$ **[1]**

***Process model***

Here $\boldsymbol{\pi}_{\boldsymbol{it}}$ (specified as a function of spatial random effects, temporal effects and spatio – temporal interaction effects) is the underlying relative risk of maternal anaemia at the sub-county $i$ at time $t$, and the is the hidden process of interest.

$$Log\left( \boldsymbol{\mu}_{it} \right)= Log\left( E_{it} \right)+Log(\boldsymbol{\pi}_{\boldsymbol{it}})$$

Where $Log\left( E_{it} \right)$is the offset and $Log\left( \boldsymbol{\pi}_{\boldsymbol{it}} \right)$ is modelled as;

$Log\left( \pi_{it} \right)= \propto+\lambda_{i}+\xi_{t}+v_{it}, i=1,\ldots,I;t=1,\ldots,T$

$\boldsymbol{\pi}_{\boldsymbol{it}}\boldsymbol{=exp(\propto+}\boldsymbol{\lambda}_{\boldsymbol{i}}\boldsymbol{+}\boldsymbol{\xi}_{\boldsymbol{t}}\boldsymbol{+}\boldsymbol{v}_{\boldsymbol{it}}\boldsymbol{)}$ **[2]**

Where $\boldsymbol{\propto}$ is the global risk, $\boldsymbol{\lambda}_{\boldsymbol{i}}$ is the main spatial effects, $\boldsymbol{\xi}_{\boldsymbol{t}}$ is the main temporal effects, and space-time interaction terms is represented by $\boldsymbol{v}_{\boldsymbol{it}}$**.** The random effects ($\lambda_{i}, \xi_{t}$) were assigned prior distributions across the space-time cube to better capture the underlying structure of maternal anaemia prevalence.

The spatial dependence was represented by the neighborhood matrix defining for each sub-county $i$ , its set of adjacent neighbours denoted by $\delta_{i}$. The neighbours are defined in terms of sub-counties sharing at least a common boundary (queen adjacency). The adjacency matrix $\boldsymbol{W}$ was then used to characterize the spatial relationship between the sub-counties.

i.e. $\boldsymbol{W=}(\omega_{ij})$, where $\omega_{ij}=1$ if we consider $i$ and $j$ to be adjacent and 0 otherwise[1].

Assuming a Besag – York – Mollie (BYM) specification, the spatial dependency $\omega_{ij}$ was formalized using an intrinsic conditional autoregressive structured model (ICAR)[2-4].

$\omega_{ij}|\omega_{ij\neq ij}\sim N(\frac{1}{N_{i}}\sum_{i} \omega_{ij}, \frac{1}{\tau_{\omega_{i}}})$ **[3]**

Where $\tau_{\omega_{i}}$ was the precision parameter and $N_{i}$ was the number of neighbours of sub-county $i$. Thus, the effect of $\omega_{ij}$ for each sub-county $i$ was normally distributed.

Then for a given set of parameters $\mu_{i}$,

$p\left( \mu_{i} | \mu_{j}, j\neq i \right)\sim N(\bar{\mu}_{i},\frac{\sigma_{\mu}^{2}}{k_{i}})$ **[4]**

Where ${\sigma^{2}}_{\mu}$ is an unknown variance parameter, $\bar{\mu}_{i}=\sum_{j\epsilon\delta_{i}} \frac{\mu_{j}}{k_{i}}$ and $k_{i}$ is the number of neighbours of area $i$. Thus, parameter values in one area was influenced by the average of its neighbours. Additional variability was quantified by the conditional variant $\frac{\sigma_{\mu}^{2}}{k_{i}}$.

The notation $\mu_{i}\sim CAR(\boldsymbol{W}, {\sigma^{2}}_{\mu})$ represents the process specified in [4].

The CAR model assumes strong dependence and has one free parameter linked to the conditional variance ${\sigma^{2}}_{\mu}$. To enhance model flexibility, we use as spatial prior the sum of the CAR process and an unstructured exchangeable normal component with mean 0 and variance ${\sigma^{2}}_{\lambda}$. The model formulated as specified in equation [5] (Convolution BYM model)

$\lambda_{i}\sim N\left( \mu_{i},{\sigma^{2}}_{\lambda} \right), i=1,\ldots, 290$; $\mu_{i}\sim CAR(\boldsymbol{W}, {\sigma^{2}}_{\mu})$. **[5]**

The CAR model was also used to model the temporal dependence[5]. Two adjacent period points (preceding and post) are defined as neighbours and then implemented using the period adjacency matrix $\boldsymbol{Q.}$ This is specified in equation [6].

$\xi_{t}\sim N\left( \gamma_{t},{\sigma^{2}}_{\xi} \right), t=1,\ldots, 3;$ $\gamma_{t}\sim CAR(Q, {\sigma^{2}}_{\gamma})$ **[6]**

The space-time interactions parameters $\boldsymbol{[v}_{\boldsymbol{it}}\boldsymbol{=}i=1,\ldots,290;t=1,\ldots,16]$ accounted for the departure from the predictable prevalence patterns based on the overall spatial risk surface and overall temporal trend. The temporal and spatial effects characterize the stability of the underlying spatial patterns. Large fluctuations indicate prevalence instability in a given sub-county *i.* The space-time random effect primarily distinguishes stable predictable patterns to accommodate model flexibility by allowing certain sub-counties to have true departures from the spatial and temporal effects.

***Parameter model***

Here the joint prior distribution of the parameters in the data level and process level are specified. The overdispersion parameter ($\boldsymbol{r}$) assumed a Gaussian prior with mean 0 and precision 1x10^-4^.

i.e. $\boldsymbol{r}\sim N\left( 0,{10}^{4} \right)$

Gamma hyperprior distribution with shape parameters 0.5 and inverse scale parameters 0.0005 were specified for the inverse variances (precision) of the spatial and temporal random effects[6].

i.e. $\tau_{\mu}=\frac{1}{{\sigma^{2}}_{\mu}},\tau_{\xi}= \frac{1}{{\sigma^{2}}_{\xi}},\tau_{\gamma}= \frac{1}{{\sigma^{2}}_{\gamma}}\sim Gamma(0.5, 0.0005)$

**Model convergence**

A two-chain Markov Chain Monte Carlo simulation was used to estimate the model parameters. Model convergence was assessed by both observing the trace and density plots of each parameter and analytically by the Gelman-Rubin statistics[7]. A MC error/SD of less than 5% pointed to the model stability/convergence.

**Supplementary Table 1: Parameter posterior distribution­ with Monte Carlo (MC) error divided by the standard deviation (SD)**

| **Node** | **mean** | **SD** | **MC error** | **2.50%** | **median** | **97.50%** | **start** | **sample** | **MC error/SD (%)** |
| --- | --- | --- | --- | --- | --- | --- | --- | --- | --- |
| **beta0** | -0.4302 | 0.01988 | 0.00093 | -0.4578 | -0.4319 | -0.3914 | 4001 | 71000 | 4.68% |
| **kappa** | 1.5210 | 0.66260 | 0.02271 | 1.2080 | 1.4120 | 2.8030 | 4001 | 71000 | 3.43% |
| **sigma.nu[1]** | 0.0284 | 0.03656 | 0.00101 | 0.0075 | 0.0273 | 0.0628 | 4001 | 71000 | 2.76% |
| **sigma.nu[2]** | 1.5490 | 0.47720 | 0.02329 | 1.2230 | 1.4380 | 2.8640 | 4001 | 71000 | 4.88% |
| **sigma.t** | 0.4211 | 0.08946 | 0.00228 | 0.2794 | 0.4098 | 0.6283 | 4001 | 71000 | 2.55% |
| **sigma.w** | 1.2140 | 0.05735 | 0.00114 | 1.1060 | 1..2130 | 1.3310 | 4001 | 71000 | 1.99% |

**Model validation:**

The fidelity of the model was assessed by a random sample of 500 data points were drawn from the space-time cube. The data with the removed points were then re-inputted into WinBUGS. To ascertain the predictive power of the model the posterior distribution of the data points was then compared with the observed values.


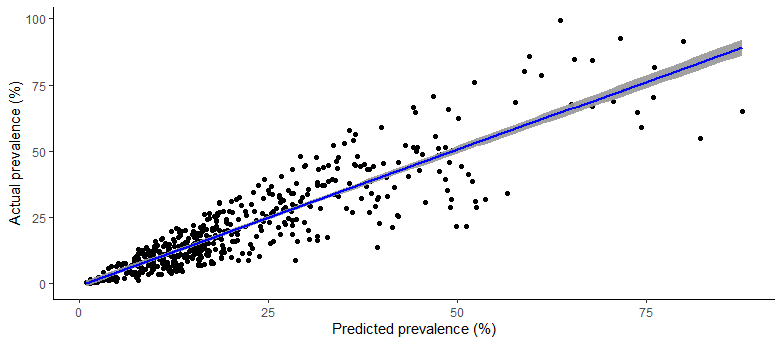


**Figure 1: Model validation.** Predicted prevalence versus observed prevalence for 500 randomly selected data points. Ninety-nine per cent of data points were within 95% credible interval (CI); Pearson’s product moment correlation 0.896 (95% UI, 0.878 - 0.912), P < 0.001 (two-sided test).

**Gelman – Rubin diagnostic check**

**Figure 2: Model convergence**: Gelman-Rubin-Brooks diagnostics plots demonstrating convergence during MCMC simulation for key model parameters. Red line represents ratio of within chain variability to between chain variability, the blue line represents the within-chain variability and the green line represents the between-chain variability (average).

**Winbugs code**

MODEL

{

for( i in 1 : N ) {

for( j in 1 : T ) {

# - Space-time interaction

nu[i,j] ~ dnorm(0, tau.nu[ind[i,j]])

#Likelihood

hb[i , j] ~ dnegbin(p[i,j],r)

p[i,j] <-r/(r+mu[i,j])

log(mu[i,j])<-log(E[i,j])+beta0+w[i]+theta[j]+nu[i,j]

#rr[i,j] <- mu[i,j]/E[i,j] ##relative risk

}

}

#- Space

w[1:N]~car.normal(adj[],weights[],num[],tau.w)

for(k in 1:sumNumNeigh){

weights[k]<-1

}

# - Time:

theta[1:T] ~ car.normal(adj.t[], weights.t[], num.t[], tau.t)

for(t in 1:1) {

weights.t[t] <- 1;

adj.t[t] <- t+1;

num.t[t] <- 1

}

for(t in 2:(T-1)) {

weights.t[2+(t-2)*2] <- 1;

adj.t[2+(t-2)*2] <- t-1

weights.t[3+(t-2)*2] <- 1;

adj.t[3+(t-2)*2] <- t+1;

num.t[t] <- 2

}

for(t in T:T) {

weights.t[(T-2)*2 + 2] <- 1;

adj.t[(T-2)*2 + 2] <- t-1;

num.t[t] <- 1

}

# - Interaction

for(i in 1:N){

sd.nu[i] <- sd(nu[i, 1:T])

}

for(i in 1:N){

for(j in 1:T){

ind[i,j] ~ dcat(P[])

}

}

sigma.nu[1] ~ dnorm(0, 100)I(0.0,)

kappa ~ dnorm(0, 0.1)I(0.0,)

sigma.nu[2] <- sigma.nu[1] + kappa

P[1:2] ~ ddirch(alpha[])

for(i in 1:2){

tau.nu[i] <- pow(sigma.nu[i], -2)

}

alpha[1] <- 1

alpha[2] <- 1

#Hyper priors

tau.t ~ dgamma(0.5,0.0005)

tau.w ~ dgamma(0.5, 0.0005)

sigma.t <- 1/sqrt(tau.t)

sigma.w <- 1/sqrt(tau.w)

# Dispersion

r ~ dgamma(1,0.1)

# Constant and coefficients

beta0 ~ dnorm(0,0.1)

}

**List of references**

1. Besag J, Green PJ: **Spatial statistics and Bayesian computation**. *Journal of the Royal Statistical Society: Series B (Methodological)* 1993, **55**(1):25-37.

2. Besag J: **Spatial interaction and the statistical analysis of lattice systems**. *Journal of the Royal Statistical Society: Series B (Methodological)* 1974, **36**(2):192-225.

3. Besag J, York J, Mollié A: **Bayesian image restoration, with two applications in spatial statistics**. *Annals of the institute of statistical mathematics* 1991, **43**(1):1-20.

4. Lawson AB, Biggeri A, Böhning D, Lesaffre E, Viel J, Clark A, Schlattmann P, Divino F: **Disease mapping models: an empirical evaluation. Disease Mapping Collaborative Group**. *Statistics in medicine* 2000, **19**(17-18):2217-2241.

5. Snow RW, Sartorius B, Kyalo D, Maina J, Amratia P, Mundia CW, Bejon P, Noor AM: **The prevalence of Plasmodium falciparum in sub-Saharan Africa since 1900**. *Nature* 2017, **550**(7677):515.

6. Kelsall J, Wakefield J: **Discussion of ‘Bayesian models for spatially correlated disease and exposure data’, by Best et al**. *Bayesian statistics* 1999, **6**:151.

7. Raftery AE, Lewis SM: **[Practical Markov Chain Monte Carlo]: comment: one long run with diagnostics: implementation strategies for Markov Chain Monte Carlo**. *Statistical science* 1992, **7**(4):493-497.
