# Supplementary material for: Mapping of anaemia prevalence among pregnant women in Kenya (2016–2019)
Source: BMC Pregnancy Childbirth. 2020 Nov 23;20:711. doi: 10.1186/s12884-020-03380-2 (PMC7685542; doi:10.1186/s12884-020-03380-2)
Supplement: Supplementary file 2 — Additional file 2. Additional data descriptions, methodological information and results. [file 12884_2020_3380_MOESM2_ESM.docx]

**Additional data descriptions, methodological information and results**

**Table 1: List of sub-counties (numbered) and their corresponding county and malaria endemicity as presented in Figure 1**

| **Endemicity** | **County** | **Sub-county** |
| --- | --- | --- |
| **Coast endemic** | Mombasa, Kwale, Kilifi, Taita Taveta | Changamwe [1], Jomvu[2], Kisauni[3], Nyali[4], Likoni[5], Mvita[6], Msambweni[7], Lunga Lunga[8], Matuga[9], Kinango[10], Kilifi North[11], Kilifi South[12], Kaloleni[13], Rabai[14], Ganze[15], Malindi[16], Magarini[17], Lamu East[21], Lamu West[22], Taveta[23], Wundanyi[24], Mwatate[25], Voi[26]. |
| **Highland** | West Pokot, Trans Nzoia, Uasin Gishu, Nandi, Baringo, Narok, Kericho, Bomet, Kisii, Nyamira | Kapenguria[129], Sigor[130], Kacheliba[131], Pokot South[132], Kwanza[136], Endebess[137], Saboti[138], Kiminini[139], Cherangany[140], Soy[141], Turbo[142], Moiben[143], Ainabkoi[144], Kapseret[145], Kesses[146], Tinderet[151], Aldai[152], Nandi Hills[153], Chesumei[154], Emgwen[155], Mosop[156], Tiaty[157], Baringo North[158], Baringo Central[159], Baringo South[160], Mogotio[161], Eldama Ravine[162], Kilgoris[177], Emurua Dikirr[178], Narok West[179], Narok North[180], Narok East[181], Narok South[182], Kipkelion East[188], Kipkelion West[189], Ainamoi[190], Bureti[191], Belgut[192], Sigowet/Soin[193], Sotik[194], Chepalungu[195], Bomet East[196], Bomet Central[197], Konoin[198], Bonchari[261], South Mugirango[262], Bomachoge Borabu[263], Bobasi[264], Bomachoge Chache[265], Nyaribari Masaba[266], Nyaribari Chache[267], Kitutu Chache North[268], Kitutu Chache South[269], Kitutu Masaba[270], West Mugirango[271], North Mugirango[272], Borabu[273]. |
| **Lake endemic** | Kakamega, Vihiga, Bungoma, Busia, Siaya, Kisumu, Homa Bay, Migori | Lugari[199], Likuyani[200], Malava[201], Lurambi[202], Navakholo[203], Mumias West[204], Mumias East[205], Matungu[206], Butere[207], Khwisero[208], Shinyalu[209], Ikolomani[210], Vihiga[211], Sabatia[212], Hamisi[213], Luanda[214], Emuhaya[215], Mt. Elgon[216], Sirisia[217], Kabuchai[218], Bumula[219], Kanduyi[220], Webuye East[221], Webuye West[222], Kimilili[223], Tongaren[224], Teso North[225], Teso South[226], Nambale[227], Matayos[228], Butula[229], Funyula[230], Budalangi[231], Ugenya[232], Ugunja[233], Alego Usonga[234], Gem[235], Bondo[236], Rarieda[237], Kisumu East[238], Kisumu West[239], Kisumu Central[240], Seme[241], Nyando[242], Muhoroni[243], Nyakach[244], Kasipul[245], Kabondo Kasipul[246], Karachuonyo[247], Rangwe[248], Homa Bay Town[249], Ndhiwa[250], Suba North[251], Suba South[252], Rongo[253], Awendo[254], Suna East[255], Suna West[256], Uriri[257], Nyatike[258], Kuria West[259], Kuria East[260]. |
| **Low risk** | Machakos, Makueni, Nyandarua, Nyeri, Kirinyaga, Muranga, Kiambu, Laikipia, Nakuru, Nairobi | Masinga[75], Yatta[76], Kangundo[77], Matungulu[78], Kathiani[79], Mavoko[80], Machakos Town[81], Mwala[82], Mbooni[83], Kilome[84], Kaiti[85], Makueni[86], Kibwezi West[87], Kibwezi East[88], Kinangop[89], Kipipiri[90], Olkalou[91], Ol Jorok[92], Ndaragwa[93], Tetu[94], Kieni[95], Mathira[96], Othaya[97], Mukurweini[98], Nyeri Town[99], Mwea[100], Gichugu[101], Ndia[102], Kirinyaga Central[103], Kangema[104], Mathioya[105], Kiharu[106], Kigumo[107], Maragwa[108], Kandara[109], Gatanga[110], Gatundu South[111], Gatundu North[112], Juja[113], Thika Town[114], Ruiru[115], Githunguri[116], Kiambu Town[117], Kiambaa[118], Kabete[119], Kikuyu[120], Limuru[121], Lari[122], Laikipia West[163], Laikipia East[164], Laikipia North[165], Molo[166], Njoro[167], Naivasha[168], Gilgil[169], Kuresoi South[170], Kuresoi North[171], Subukia[172], Rongai[173], Bahati[174], Nakuru West[175], Nakuru East[176], Westlands[274], Kilimani[275], Dagoretti[276], Langata[277], Kibra[278], Roysambu[279], Kasarani[280], Ruaraka[281], Embakasi South[282], Embakasi North[283], Embakasi Central[284], Embakasi East[285], Embakasi West[286], Makadara[287], Kamukunji[288], Starehe[289], Mathare[290]. |
| **Seasonal** | Tana River, Garissa, Wajir, Mandera, Marsabit, Isiolo, Meru, Tharaka-Nithi, Embu, Kitui, Turkana, Samburu, Elgeyo-Marakwet, Kajiado | Garsen[18], Galole[19], Bura[20], Dujis[27], Balambala[28], Lagdera[29], Dadaab[30], Fafi[31], Ijara[32], Wajir North[33], Wajir East[34], Tarbaj[35], Wajir West[36], Eldas[37], Wajir South[38], Mandera West[39], Banissa[40], Mandera North[41], Mandera South[42], Mandera East[43], Lafey[44], Moyale[45], North Horr[46], Saku[47], Laisamis[48], Isiolo North[49], Isiolo South[50], Igembe South[51], Igembe Central[52], Igembe North[53], Tigania West[54], Tigania East[55], North Imenti[56], Buuri[57], Central Imenti[58], South Imenti[59], Nithi[60], Maara[61], Tharaka[62], Manyatta[63], Runyenjes[64], Gachoka[65], Siakago[66], Mwingi North[67], Mwingi West[68], Mwingi East[69], Kitui West[70], Kitui Rural[71], Kitui Central[72], Kitui East[73], Kitui South[74], Turkana North[123], Turkana West[124], Turkana Central[125], Loima[126], Turkana South[127], Turkana East[128], Samburu Central[133], Samburu North[134], Samburu East[135], Marakwet East[147], Marakwet West[148], Keiyo North[149], Keiyo South[150], Kajiado North[183], Kajiado Central[184], Kajiado West[185], Kajiado East[186], Kajiado South[187]. |

**Table 2: Number of sub-counties categorized by public health significance of anaemia (quarterly and yearly)**

| **Public Health Significance** | **Quarter** | **Year** | | | |
| --- | --- | --- | --- | --- | --- |
|  |  | **2016** | **2017** | **2018** | **2019** |
| **Normal (<5.0%)** | Jan - Mar | 215 | 30 | 25 | 15 |
|  | Apr - Jun | 52 | 32 | 25 | 16 |
|  | Jul - Sept | 34 | 46 | 22 | 18 |
|  | Oct - Dec | 24 | 31 | 26 | 14 |
| **Mild (5.0 – 19.9%)** | Jan - Mar | 70 | 168 | 164 | 152 |
|  | Apr - Jun | 178 | 162 | 156 | 133 |
|  | Jul - Sept | 157 | 158 | 151 | 125 |
|  | Oct - Dec | 154 | 170 | 148 | 145 |
| **Moderate (20.0 – 39.9%)** | Jan - Mar | 4 | 60 | 63 | 80 |
|  | Apr - Jun | 50 | 59 | 67 | 92 |
|  | Jul - Sept | 69 | 66 | 75 | 94 |
|  | Oct - Dec | 72 | 56 | 73 | 83 |
| **Severe (>40.0%)** | Jan - Mar | 1 | 32 | 38 | 43 |
|  | Apr - Jun | 10 | 37 | 42 | 49 |
|  | Jul - Sept | 30 | 20 | 42 | 53 |
|  | Oct - Dec | 40 | 33 | 43 | 48 |

In 2016 the proportion of sub-counties with normal prevalence decreased from 66.2% (95% UI: 61.0 – 71.3) in the first quarter to 9.3% (95% UI: 4.5 – 10.2) in the fourth quarter. Over the same period; mild prevalence increased from 12.5% (95% UI: 9.8 – 15.3) to 27.6% (95% UI: 23.9 – 31.3), moderate prevalence increased from 2.1% (95% UI: 0.1 – 4.0) to 36.9% (95% UI: 30.2 – 43.7) and severe prevalence also increased from 1.2% (95% UI: 1.1 – 3.6) to 49.4 (38.5 – 60.3). In 2017, maternal anaemia prevalence estimates ranged from 17.0 % (16.8 – 17.2) in the third quarter to 21.7% (21.5 – 21.8) in the first quarter. Compared to the 2016 estimates, the proportion of sub-counties with normal prevalence appears to have decreased by 86.1% (81.4 – 90.7) and by 38.5% (25.2 – 51.7) in the first and second quarter respectively. Sub-counties with severe prevalence increased by 33.3% (16.5 – 50.2) and 17.5% (5.7 – 29.3) in the third and fourth quarter respectively. In 2018, maternal anaemia prevalence estimates ranged from 22.2 % (95% UI: 22.0 – 22.3) in the first quarter to 24.3% (95% UI: 24.1 – 24.5) in the fourth quarter. with the proportion of sub-counties with normal prevalence and mild prevalence thresholds decreasing to 8.5% (95% UI: 6.9 – 10.1) and 53.4% (95% UI: 50.5 – 56.2) respectively. In 2019, the estimated maternal anaemia prevalence ranged from 24.6 % (24.4 – 24.7) in the first quarter to 27.6% (27.5 – 27.8) in the third quarter. Additionally, the proportion of sub-counties with normal prevalence was 5.4% (4.1 – 6.7), mild prevalence was 52.2% (45.0 – 50.7), moderate prevalence was 30.1% (27.5 – 32.7) and severe prevalence was 16.6% (14.5 – 18.8). Compared to the 2018 estimates, sub-counties with normal and mild prevalence decreased by 35.7% (26.2 – 45.2) and 10.3% (7.9 – 12.7) respectively. On the other hand, sub-counties with moderate and severe prevalence increased by 25.5% (20.4 – 30.7) and 17.0% (11.2 – 22.7)

**Table 2: Posterior median estimates for each sub-county between 2016 – 2019 stratified by malaria endemicity**

|  |  |  |  | **2016** | | | | **2017** | | | | | **2018** | | | | | **2019** | | | | |  |
| --- | --- | --- | --- | --- | --- | --- | --- | --- | --- | --- | --- | --- | --- | --- | --- | --- | --- | --- | --- | --- | --- | --- | --- |
| **ID** | **Endemicity** | **County** | **Sub County** | **Q1** | **Q2** | **Q3** | **Q4** | | **Q1** | **Q2** | **Q3** | **Q4** | | **Q1** | **Q2** | **Q3** | **Q4** | | **Q1** | **Q2** | **Q3** | **Q4** | |
| 1 | Coast endemic | Mombasa | Changamwe | 7.2 | 31.9 | 42.8 | 49.7 | | 43.9 | 45.3 | 38.4 | 43.3 | | 48.0 | 49.7 | 50.8 | 52.1 | | 53.1 | 57.7 | 58.9 | 55.1 | |
| 2 | Coast endemic | Mombasa | Jomvu | 8.6 | 37.5 | 49.0 | 52.6 | | 49.4 | 52.2 | 44.4 | 49.3 | | 53.4 | 54.5 | 55.4 | 56.9 | | 58.6 | 63.4 | 64.9 | 62.6 | |
| 3 | Coast endemic | Mombasa | Kisauni | 13.7 | 44.9 | 61.5 | 68.2 | | 61.2 | 64.8 | 55.6 | 60.5 | | 66.2 | 69.1 | 70.4 | 72.3 | | 74.7 | 81.1 | 83.2 | 77.1 | |
| 4 | Coast endemic | Mombasa | Nyali | 11.3 | 49.3 | 66.2 | 73.2 | | 65.8 | 69.8 | 57.2 | 64.1 | | 70.8 | 73.3 | 75.4 | 76.8 | | 78.0 | 86.3 | 86.0 | 82.5 | |
| 5 | Coast endemic | Mombasa | Likoni | 8.4 | 37.9 | 50.6 | 56.4 | | 50.6 | 53.7 | 43.8 | 51.3 | | 55.4 | 56.5 | 58.1 | 59.3 | | 60.9 | 66.2 | 66.8 | 63.8 | |
| 6 | Coast endemic | Mombasa | Mvita | 7.2 | 33.1 | 44.8 | 50.7 | | 46.5 | 48.4 | 40.5 | 42.9 | | 48.7 | 50.6 | 51.7 | 52.7 | | 55.0 | 59.2 | 59.4 | 56.8 | |
| 7 | Coast endemic | Kwale | Msambweni | 13.1 | 47.4 | 80.7 | 90.1 | | 80.4 | 85.1 | 66.0 | 76.6 | | 90.4 | 92.0 | 94.0 | 97.7 | | 91.5 | 87.0 | 88.5 | 93.0 | |
| 8 | Coast endemic | Kwale | Lunga Lunga | 7.2 | 34.2 | 46.4 | 52.2 | | 46.4 | 49.2 | 35.8 | 43.9 | | 50.9 | 51.7 | 53.2 | 54.5 | | 55.5 | 60.3 | 61.3 | 60.2 | |
| 9 | Coast endemic | Kwale | Matuga | 11.1 | 56.7 | 70.9 | 77.3 | | 70.0 | 78.1 | 61.7 | 68.0 | | 76.0 | 79.2 | 79.2 | 81.8 | | 83.1 | 77.7 | 90.4 | 85.9 | |
| 10 | Coast endemic | Kwale | Kinango | 4.7 | 35.6 | 50.5 | 56.2 | | 50.4 | 53.9 | 44.1 | 48.3 | | 54.2 | 58.0 | 57.1 | 60.7 | | 60.1 | 65.1 | 65.5 | 62.4 | |
| 11 | Coast endemic | Kilifi | Kilifi North | 7.0 | 30.9 | 40.7 | 45.7 | | 40.9 | 43.2 | 38.0 | 39.3 | | 43.7 | 45.4 | 47.8 | 48.2 | | 49.3 | 53.8 | 54.0 | 51.2 | |
| 12 | Coast endemic | Kilifi | Kilifi South | 6.4 | 33.0 | 43.8 | 48.2 | | 43.3 | 45.2 | 35.0 | 41.6 | | 46.8 | 48.5 | 49.5 | 51.7 | | 52.0 | 57.0 | 57.5 | 54.2 | |
| 13 | Coast endemic | Kilifi | Kaloleni | 6.2 | 30.1 | 42.8 | 46.9 | | 41.5 | 43.2 | 36.7 | 40.1 | | 44.6 | 45.7 | 47.8 | 48.7 | | 49.6 | 54.7 | 55.1 | 52.3 | |
| 14 | Coast endemic | Kilifi | Rabai | 6.6 | 31.2 | 42.6 | 47.5 | | 42.4 | 45.2 | 39.4 | 40.8 | | 49.1 | 56.6 | 49.7 | 50.1 | | 51.6 | 56.1 | 53.9 | 52.6 | |
| 15 | Coast endemic | Kilifi | Ganze | 17.1 | 33.1 | 40.9 | 46.0 | | 43.3 | 43.9 | 35.7 | 39.8 | | 44.8 | 45.8 | 46.9 | 47.3 | | 49.7 | 54.2 | 54.4 | 51.6 | |
| 16 | Coast endemic | Kilifi | Malindi | 5.9 | 33.6 | 46.9 | 52.5 | | 47.2 | 49.9 | 44.9 | 45.8 | | 50.0 | 52.7 | 54.1 | 55.8 | | 57.3 | 61.8 | 63.1 | 59.2 | |
| 17 | Coast endemic | Kilifi | Magarini | 7.4 | 38.0 | 51.6 | 57.0 | | 49.5 | 53.0 | 40.2 | 47.3 | | 53.4 | 56.2 | 57.2 | 58.4 | | 60.1 | 65.1 | 66.4 | 61.8 | |
| 18 | Seasonal | Tana River | Garsen | 8.6 | 38.6 | 52.3 | 58.5 | | 52.4 | 56.2 | 47.5 | 50.3 | | 55.8 | 58.0 | 58.7 | 59.2 | | 62.4 | 68.3 | 68.0 | 64.6 | |
| 19 | Seasonal | Tana River | Galole | 47.8 | 47.5 | 54.2 | 59.1 | | 53.3 | 57.1 | 33.7 | 50.4 | | 58.2 | 59.1 | 60.1 | 60.7 | | 64.3 | 68.4 | 68.7 | 67.0 | |
| 20 | Seasonal | Tana River | Bura | 9.0 | 28.9 | 39.2 | 43.4 | | 39.0 | 41.6 | 38.4 | 37.3 | | 42.2 | 44.0 | 45.0 | 43.6 | | 46.0 | 52.3 | 51.1 | 48.3 | |
| 21 | Coast endemic | Lamu | Lamu East | 9.0 | 33.7 | 50.8 | 55.2 | | 49.0 | 50.3 | 36.2 | 46.0 | | 50.9 | 52.3 | 53.7 | 54.6 | | 56.3 | 58.1 | 60.6 | 58.9 | |
| 22 | Coast endemic | Lamu | Lamu West | 14.6 | 44.1 | 60.7 | 67.7 | | 60.6 | 64.6 | 51.7 | 59.4 | | 64.6 | 66.6 | 68.2 | 70.5 | | 71.0 | 80.1 | 78.1 | 74.7 | |
| 23 | Coast endemic | Taita Taveta | Taveta | 4.2 | 19.9 | 28.3 | 31.1 | | 28.2 | 30.0 | 23.8 | 28.0 | | 29.2 | 29.5 | 32.8 | 33.0 | | 34.0 | 36.7 | 36.7 | 35.1 | |
| 24 | Coast endemic | Taita Taveta | Wundanyi | 2.1 | 5.1 | 15.9 | 18.2 | | 15.9 | 16.2 | 40.2 | 14.9 | | 17.2 | 17.8 | 18.2 | 18.9 | | 19.1 | 21.1 | 21.3 | 20.8 | |
| 25 | Coast endemic | Taita Taveta | Mwatate | 2.1 | 12.6 | 23.2 | 26.0 | | 23.5 | 24.5 | 20.4 | 22.4 | | 16.9 | 24.9 | 28.1 | 28.3 | | 28.2 | 31.6 | 31.0 | 29.6 | |
| 26 | Coast endemic | Taita Taveta | Voi | 5.4 | 25.0 | 34.4 | 39.2 | | 35.1 | 37.1 | 30.6 | 35.3 | | 37.8 | 38.8 | 40.1 | 41.2 | | 42.6 | 45.9 | 47.1 | 43.7 | |
| 27 | Seasonal | Garissa | Dujis | 5.1 | 22.9 | 30.1 | 33.6 | | 30.0 | 30.2 | 29.5 | 29.6 | | 33.3 | 36.2 | 37.6 | 39.4 | | 39.3 | 42.7 | 43.1 | 43.3 | |
| 28 | Seasonal | Garissa | Balambala | 11.0 | 23.8 | 35.2 | 36.0 | | 34.1 | 34.8 | 24.0 | 32.0 | | 35.7 | 37.5 | 40.7 | 39.7 | | 40.5 | 44.6 | 46.7 | 42.8 | |
| 29 | Seasonal | Garissa | Lagdera | 7.3 | 23.6 | 31.1 | 33.8 | | 32.3 | 32.3 | 25.4 | 27.7 | | 33.0 | 35.5 | 35.7 | 39.8 | | 37.8 | 40.6 | 41.5 | 38.8 | |
| 30 | Seasonal | Garissa | Dadaab | 2.7 | 13.7 | 15.7 | 21.1 | | 21.2 | 19.8 | 16.6 | 17.6 | | 20.6 | 21.0 | 22.8 | 21.6 | | 24.0 | 25.8 | 25.0 | 23.7 | |
| 31 | Seasonal | Garissa | Fafi | 6.7 | 20.8 | 42.4 | 47.7 | | 43.9 | 44.9 | 37.0 | 41.9 | | 44.4 | 48.1 | 49.3 | 51.2 | | 50.3 | 55.5 | 56.1 | 53.1 | |
| 32 | Seasonal | Garissa | Ijara | 15.0 | 18.7 | 25.2 | 26.8 | | 25.2 | 26.4 | 21.4 | 14.5 | | 26.9 | 28.9 | 30.4 | 29.8 | | 30.0 | 33.1 | 34.7 | 31.3 | |
| 33 | Seasonal | Wajir | Wajir North | 9.9 | 26.5 | 32.5 | 36.9 | | 36.7 | 34.4 | 28.3 | 31.6 | | 35.5 | 38.3 | 36.4 | 35.9 | | 34.5 | 33.1 | 41.9 | 37.4 | |
| 34 | Seasonal | Wajir | Wajir East | 14.9 | 12.8 | 13.5 | 15.1 | | 16.3 | 17.6 | 13.3 | 14.8 | | 16.5 | 48.3 | 16.9 | 18.3 | | 13.3 | 19.9 | 21.1 | 19.5 | |
| 35 | Seasonal | Wajir | Tarbaj | 6.1 | 5.5 | 7.5 | 7.8 | | 7.1 | 7.2 | 6.1 | 6.7 | | 7.6 | 7.7 | 7.0 | 3.9 | | 8.1 | 9.1 | 9.7 | 9.0 | |
| 36 | Seasonal | Wajir | Wajir West | 6.3 | 29.1 | 39.0 | 44.9 | | 40.7 | 43.1 | 34.8 | 40.5 | | 43.1 | 44.4 | 45.5 | 43.4 | | 44.6 | 52.2 | 52.8 | 50.2 | |
| 37 | Seasonal | Wajir | Eldas | 3.6 | 13.6 | 8.2 | 22.1 | | 19.6 | 23.8 | 16.1 | 18.9 | | 21.5 | 19.4 | 21.6 | 20.1 | | 5.2 | 23.2 | 26.1 | 23.9 | |
| 38 | Seasonal | Wajir | Wajir South | 6.3 | 15.7 | 19.9 | 24.3 | | 21.3 | 22.1 | 19.4 | 20.4 | | 22.5 | 23.5 | 23.1 | 24.9 | | 24.8 | 26.8 | 28.1 | 26.1 | |
| 39 | Seasonal | Mandera | Mandera West | 2.1 | 7.7 | 2.6 | 5.7 | | 10.3 | 9.2 | 10.9 | 12.4 | | 17.3 | 15.1 | 22.7 | 15.9 | | 14.4 | 16.5 | 16.5 | 14.8 | |
| 40 | Seasonal | Mandera | Banissa | 4.0 | 3.0 | 25.5 | 28.6 | | 7.5 | 22.8 | 18.4 | 23.7 | | 12.8 | 23.6 | 25.9 | 25.1 | | 25.4 | 28.2 | 26.9 | 26.2 | |
| 41 | Seasonal | Mandera | Mandera North | 1.2 | 2.4 | 1.8 | 4.6 | | 3.1 | 9.7 | 6.0 | 4.1 | | 12.6 | 8.7 | 35.7 | 10.7 | | 8.7 | 9.9 | 9.5 | 4.2 | |
| 42 | Seasonal | Mandera | Mandera South | 2.4 | 9.6 | 14.5 | 12.5 | | 3.9 | 3.4 | 12.9 | 13.7 | | 16.2 | 25.2 | 17.4 | 18.6 | | 17.9 | 18.4 | 20.3 | 20.2 | |
| 43 | Seasonal | Mandera | Mandera East | 5.2 | 14.2 | 32.6 | 38.0 | | 33.3 | 9.4 | 35.1 | 31.8 | | 35.9 | 51.3 | 38.6 | 26.4 | | 40.1 | 48.3 | 60.6 | 57.5 | |
| 44 | Seasonal | Mandera | Lafey | 10.0 | 24.9 | 31.8 | 44.6 | | 44.6 | 51.8 | 30.3 | 35.4 | | 36.9 | 40.8 | 39.8 | 38.9 | | 13.5 | 34.3 | 27.0 | 40.6 | |
| 45 | Seasonal | Marsabit | Moyale | 5.9 | 30.5 | 41.3 | 46.3 | | 42.0 | 46.2 | 27.9 | 40.3 | | 45.6 | 47.7 | 47.9 | 48.8 | | 51.1 | 54.5 | 57.2 | 53.3 | |
| 46 | Seasonal | Marsabit | North Horr | 1.6 | 7.1 | 9.5 | 10.8 | | 9.5 | 9.7 | 7.9 | 9.3 | | 10.0 | 10.3 | 9.9 | 9.5 | | 11.1 | 12.9 | 12.6 | 11.8 | |
| 47 | Seasonal | Marsabit | Saku | 24.5 | 27.7 | 37.1 | 41.8 | | 37.7 | 39.7 | 36.3 | 38.3 | | 41.1 | 41.8 | 43.5 | 44.4 | | 45.0 | 49.1 | 49.2 | 46.5 | |
| 48 | Seasonal | Marsabit | Laisamis | 3.3 | 9.2 | 13.8 | 14.9 | | 13.0 | 13.9 | 12.6 | 12.1 | | 13.2 | 14.1 | 13.1 | 14.5 | | 10.7 | 14.8 | 16.6 | 15.4 | |
| 49 | Seasonal | Isiolo | Isiolo North | 3.7 | 11.1 | 15.0 | 17.5 | | 14.2 | 16.0 | 12.5 | 14.0 | | 16.5 | 17.3 | 17.9 | 17.8 | | 18.3 | 20.5 | 20.4 | 20.0 | |
| 50 | Seasonal | Isiolo | Isiolo South | 2.6 | 8.6 | 12.3 | 15.5 | | 12.6 | 13.1 | 11.9 | 12.0 | | 15.4 | 15.2 | 17.2 | 16.2 | | 17.2 | 20.2 | 48.5 | 44.5 | |
| 51 | Seasonal | Meru | Igembe South | 1.2 | 7.2 | 4.0 | 5.9 | | 8.3 | 17.5 | 17.6 | 22.6 | | 22.1 | 18.3 | 20.5 | 23.1 | | 20.5 | 22.2 | 22.6 | 21.2 | |
| 52 | Seasonal | Meru | Igembe Central | 0.5 | 1.7 | 3.1 | 1.8 | | 2.2 | 3.3 | 6.3 | 2.9 | | 1.4 | 2.8 | 2.8 | 3.0 | | 3.1 | 3.3 | 3.4 | 3.2 | |
| 53 | Seasonal | Meru | Igembe North | 3.3 | 12.1 | 17.5 | 6.4 | | 3.2 | 4.4 | 0.0 | 15.4 | | 18.4 | 19.1 | 19.5 | 20.2 | | 23.6 | 22.4 | 23.9 | 27.7 | |
| 54 | Seasonal | Meru | Tigania West | 1.2 | 9.7 | 13.1 | 10.2 | | 12.6 | 13.8 | 14.3 | 12.4 | | 13.7 | 14.5 | 14.6 | 18.1 | | 17.0 | 18.8 | 19.9 | 16.5 | |
| 55 | Seasonal | Meru | Tigania East | 1.8 | 10.8 | 14.0 | 16.3 | | 14.6 | 15.6 | 73.5 | 13.8 | | 15.7 | 15.9 | 16.7 | 16.7 | | 17.3 | 19.3 | 18.9 | 17.5 | |
| 56 | Seasonal | Meru | North Imenti | 0.9 | 4.2 | 4.0 | 9.8 | | 6.1 | 6.3 | 0.0 | 5.9 | | 6.5 | 6.8 | 6.9 | 7.1 | | 7.3 | 7.8 | 8.1 | 7.6 | |
| 57 | Seasonal | Meru | Buuri | 15.9 | 29.0 | 25.2 | 18.9 | | 16.9 | 18.5 | 24.3 | 17.1 | | 18.5 | 19.7 | 19.9 | 18.8 | | 18.2 | 21.1 | 22.3 | 19.6 | |
| 58 | Seasonal | Meru | Central Imenti | 2.3 | 10.6 | 12.1 | 13.6 | | 11.6 | 12.7 | 0.0 | 11.8 | | 18.7 | 17.2 | 14.8 | 13.5 | | 14.5 | 15.6 | 15.5 | 13.2 | |
| 59 | Seasonal | Meru | South Imenti | 2.0 | 9.8 | 14.2 | 10.5 | | 13.4 | 14.0 | 0.0 | 12.7 | | 14.9 | 15.4 | 16.0 | 16.4 | | 17.5 | 19.1 | 18.7 | 19.7 | |
| 60 | Seasonal | Tharaka-Nithi | Nithi | 2.2 | 9.7 | 13.7 | 15.4 | | 2.3 | 14.7 | 11.5 | 12.2 | | 15.2 | 15.7 | 16.3 | 17.9 | | 16.5 | 17.9 | 18.1 | 17.0 | |
| 61 | Seasonal | Tharaka-Nithi | Maara | 0.9 | 7.2 | 10.1 | 11.5 | | 9.0 | 10.9 | 8.7 | 8.8 | | 10.9 | 11.5 | 11.2 | 11.6 | | 12.0 | 12.9 | 13.4 | 12.2 | |
| 62 | Seasonal | Tharaka-Nithi | Tharaka | 1.1 | 10.7 | 14.9 | 16.9 | | 14.5 | 15.4 | 31.8 | 14.5 | | 16.0 | 16.5 | 17.1 | 17.3 | | 17.9 | 19.5 | 20.5 | 25.7 | |
| 63 | Seasonal | Embu | Manyatta | 1.9 | 7.1 | 10.3 | 11.9 | | 10.2 | 11.3 | 10.3 | 9.6 | | 10.7 | 11.2 | 11.7 | 11.7 | | 12.3 | 13.0 | 13.4 | 12.8 | |
| 64 | Seasonal | Embu | Runyenjes | 2.8 | 10.4 | 15.1 | 16.8 | | 15.5 | 13.6 | 14.9 | 14.3 | | 15.5 | 16.5 | 17.2 | 17.4 | | 18.1 | 19.7 | 19.8 | 18.9 | |
| 65 | Seasonal | Embu | Gachoka | 2.2 | 5.2 | 11.6 | 24.5 | | 11.7 | 12.8 | 8.9 | 11.3 | | 12.7 | 13.0 | 13.4 | 13.6 | | 13.8 | 14.4 | 15.3 | 14.2 | |
| 66 | Seasonal | Embu | Siakago | 1.6 | 2.6 | 17.5 | 38.4 | | 19.2 | 20.7 | 31.7 | 19.2 | | 20.2 | 21.7 | 22.4 | 22.1 | | 22.8 | 25.1 | 25.3 | 23.5 | |
| 67 | Seasonal | Kitui | Mwingi North | 1.3 | 11.3 | 15.8 | 17.6 | | 15.8 | 16.4 | 12.8 | 15.4 | | 17.2 | 17.6 | 18.1 | 19.5 | | 18.9 | 21.6 | 21.1 | 19.7 | |
| 68 | Seasonal | Kitui | Mwingi West | 2.8 | 9.4 | 12.8 | 14.4 | | 12.2 | 13.4 | 10.5 | 11.8 | | 13.8 | 14.1 | 14.3 | 15.2 | | 15.6 | 16.8 | 17.0 | 16.0 | |
| 69 | Seasonal | Kitui | Mwingi East | 0.9 | 14.5 | 20.1 | 21.8 | | 20.3 | 21.5 | 17.5 | 19.7 | | 21.7 | 22.6 | 23.8 | 24.8 | | 24.7 | 26.8 | 26.7 | 25.3 | |
| 70 | Seasonal | Kitui | Kitui West | 3.2 | 12.2 | 17.0 | 22.8 | | 16.9 | 16.0 | 13.6 | 16.0 | | 17.7 | 19.1 | 18.8 | 28.7 | | 21.1 | 21.9 | 22.6 | 19.0 | |
| 71 | Seasonal | Kitui | Kitui Rural | 2.1 | 12.5 | 17.7 | 20.7 | | 17.7 | 18.5 | 5.6 | 18.0 | | 20.2 | 20.0 | 88.4 | 17.3 | | 20.9 | 23.0 | 22.8 | 19.3 | |
| 72 | Seasonal | Kitui | Kitui Central | 5.7 | 23.9 | 31.5 | 36.0 | | 31.7 | 35.6 | 27.3 | 31.6 | | 34.9 | 43.1 | 37.2 | 37.0 | | 38.5 | 41.6 | 41.5 | 38.2 | |
| 73 | Seasonal | Kitui | Kitui East | 2.4 | 9.0 | 12.7 | 14.2 | | 13.1 | 13.3 | 8.7 | 12.0 | | 13.3 | 14.1 | 14.5 | 14.8 | | 15.5 | 17.1 | 16.7 | 15.7 | |
| 74 | Seasonal | Kitui | Kitui South | 1.8 | 15.4 | 16.2 | 23.3 | | 21.9 | 22.1 | 17.8 | 20.2 | | 23.0 | 23.7 | 24.3 | 25.3 | | 26.6 | 28.3 | 28.0 | 24.2 | |
| 75 | Low risk | Machakos | Masinga | 2.5 | 12.4 | 21.7 | 17.7 | | 14.9 | 15.7 | 16.4 | 15.4 | | 16.7 | 16.9 | 17.7 | 17.6 | | 18.4 | 20.7 | 20.7 | 19.8 | |
| 76 | Low risk | Machakos | Yatta | 3.6 | 13.2 | 17.5 | 19.5 | | 17.6 | 18.5 | 15.6 | 17.0 | | 20.1 | 20.0 | 21.2 | 22.2 | | 21.6 | 23.7 | 24.4 | 24.1 | |
| 77 | Low risk | Machakos | Kangundo | 1.4 | 8.4 | 11.7 | 13.0 | | 11.6 | 11.2 | 10.6 | 11.5 | | 12.9 | 13.3 | 13.3 | 13.8 | | 14.5 | 15.6 | 15.7 | 15.4 | |
| 78 | Low risk | Machakos | Matungulu | 1.7 | 7.9 | 10.5 | 12.2 | | 10.9 | 11.2 | 12.3 | 10.7 | | 11.8 | 12.2 | 12.1 | 13.0 | | 13.7 | 14.5 | 14.7 | 14.1 | |
| 79 | Low risk | Machakos | Kathiani | 5.8 | 22.3 | 30.5 | 34.3 | | 29.4 | 32.7 | 0.0 | 29.5 | | 33.4 | 34.8 | 34.8 | 40.5 | | 36.6 | 39.9 | 40.2 | 40.3 | |
| 80 | Low risk | Machakos | Mavoko | 3.3 | 12.9 | 7.0 | 19.8 | | 7.4 | 18.7 | 15.0 | 15.3 | | 18.4 | 19.3 | 22.0 | 20.2 | | 20.9 | 23.0 | 22.8 | 13.2 | |
| 81 | Low risk | Machakos | Machakos Town | 1.2 | 9.1 | 11.9 | 13.0 | | 12.1 | 12.9 | 3.7 | 11.9 | | 14.6 | 14.0 | 14.6 | 14.8 | | 14.8 | 16.3 | 16.2 | 16.3 | |
| 82 | Low risk | Machakos | Mwala | 3.5 | 16.6 | 22.7 | 25.2 | | 22.5 | 23.6 | 23.4 | 22.2 | | 24.5 | 25.1 | 26.5 | 26.5 | | 26.7 | 30.1 | 30.6 | 28.5 | |
| 83 | Low risk | Makueni | Mbooni | 3.6 | 16.0 | 23.4 | 26.5 | | 23.6 | 24.5 | 20.1 | 23.3 | | 26.9 | 26.9 | 26.5 | 27.4 | | 28.6 | 30.7 | 31.3 | 29.5 | |
| 84 | Low risk | Makueni | Kilome | 2.6 | 16.6 | 22.6 | 23.9 | | 23.9 | 25.1 | 21.1 | 22.0 | | 24.3 | 25.5 | 25.7 | 26.2 | | 27.0 | 30.1 | 30.7 | 28.6 | |
| 85 | Low risk | Makueni | Kaiti | 3.0 | 12.1 | 16.0 | 18.0 | | 16.0 | 16.8 | 13.2 | 15.4 | | 17.1 | 17.8 | 17.9 | 19.1 | | 19.1 | 20.6 | 19.4 | 18.8 | |
| 86 | Low risk | Makueni | Makueni | 4.4 | 20.3 | 25.7 | 30.6 | | 27.9 | 28.6 | 24.4 | 26.7 | | 30.0 | 31.6 | 32.5 | 33.4 | | 34.0 | 37.4 | 40.1 | 35.8 | |
| 87 | Low risk | Makueni | Kibwezi West | 4.5 | 28.2 | 35.6 | 43.0 | | 39.5 | 40.8 | 32.9 | 40.5 | | 44.7 | 42.8 | 44.5 | 45.5 | | 47.0 | 50.3 | 51.3 | 48.6 | |
| 88 | Low risk | Makueni | Kibwezi East | 14.6 | 43.1 | 55.3 | 62.4 | | 56.1 | 57.4 | 45.8 | 53.9 | | 60.4 | 63.9 | 64.2 | 65.1 | | 66.9 | 73.7 | 73.6 | 70.4 | |
| 89 | Low risk | Nyandarua | Kinangop | 1.2 | 3.0 | 6.1 | 6.6 | | 5.7 | 6.4 | 4.7 | 6.1 | | 6.6 | 6.8 | 9.0 | 7.2 | | 7.4 | 8.0 | 8.2 | 7.7 | |
| 90 | Low risk | Nyandarua | Kipipiri | 2.1 | 9.5 | 12.9 | 2.5 | | 13.0 | 2.4 | 11.3 | 6.7 | | 11.0 | 11.8 | 11.5 | 12.6 | | 15.6 | 15.1 | 15.5 | 14.0 | |
| 91 | Low risk | Nyandarua | Olkalou | 0.5 | 1.3 | 1.6 | 4.8 | | 1.5 | 4.5 | 2.5 | 3.5 | | 3.7 | 3.9 | 4.0 | 4.7 | | 5.9 | 4.8 | 4.8 | 24.3 | |
| 92 | Low risk | Nyandarua | Ol Jorok | 0.7 | 3.0 | 2.1 | 3.8 | | 3.5 | 3.6 | 3.7 | 3.3 | | 3.5 | 3.8 | 4.1 | 4.2 | | 4.3 | 5.0 | 4.6 | 4.4 | |
| 93 | Low risk | Nyandarua | Ndaragwa | 1.5 | 5.6 | 7.7 | 8.1 | | 7.0 | 7.3 | 8.6 | 7.4 | | 8.2 | 9.1 | 9.0 | 9.2 | | 9.7 | 55.0 | 54.5 | 11.7 | |
| 94 | Low risk | Nyeri | Tetu | 3.2 | 6.3 | 7.9 | 8.3 | | 8.3 | 8.4 | 6.6 | 8.6 | | 8.6 | 8.9 | 9.3 | 8.9 | | 9.2 | 11.6 | 10.5 | 9.8 | |
| 95 | Low risk | Nyeri | Kieni | 0.6 | 4.2 | 5.3 | 6.3 | | 5.6 | 5.9 | 5.0 | 5.8 | | 6.5 | 6.5 | 6.7 | 6.9 | | 6.9 | 7.5 | 7.9 | 7.0 | |
| 96 | Low risk | Nyeri | Mathira | 0.9 | 7.6 | 9.1 | 10.5 | | 9.9 | 10.6 | 8.4 | 9.0 | | 9.5 | 10.8 | 11.2 | 11.5 | | 11.8 | 12.8 | 13.1 | 12.9 | |
| 97 | Low risk | Nyeri | Othaya | 2.7 | 10.5 | 14.1 | 16.0 | | 14.6 | 15.0 | 13.5 | 13.6 | | 15.3 | 15.3 | 13.1 | 16.3 | | 16.0 | 17.7 | 18.3 | 17.3 | |
| 98 | Low risk | Nyeri | Mukurweini | 3.4 | 16.4 | 19.5 | 20.3 | | 17.5 | 18.4 | 15.7 | 17.0 | | 19.3 | 18.8 | 17.1 | 20.7 | | 21.2 | 23.1 | 22.7 | 22.1 | |
| 99 | Low risk | Nyeri | Nyeri Town | 1.1 | 10.3 | 13.9 | 15.5 | | 14.0 | 15.0 | 12.3 | 14.5 | | 15.5 | 16.1 | 16.2 | 16.6 | | 16.9 | 18.3 | 18.7 | 18.0 | |
| 100 | Low risk | Kirinyaga | Mwea | 0.6 | 6.7 | 10.6 | 12.1 | | 10.7 | 11.3 | 10.7 | 10.4 | | 11.9 | 12.3 | 12.8 | 12.9 | | 15.2 | 14.1 | 14.3 | 13.6 | |
| 101 | Low risk | Kirinyaga | Gichugu | 2.0 | 5.8 | 10.0 | 11.5 | | 10.1 | 10.7 | 49.5 | 9.7 | | 10.9 | 11.2 | 11.6 | 11.9 | | 11.9 | 13.7 | 13.4 | 15.7 | |
| 102 | Low risk | Kirinyaga | Ndia | 2.3 | 7.6 | 11.8 | 13.4 | | 12.0 | 12.5 | 19.6 | 11.8 | | 13.0 | 13.4 | 14.2 | 14.1 | | 14.4 | 15.8 | 15.3 | 15.3 | |
| 103 | Low risk | Kirinyaga | Kirinyaga Central | 1.2 | 3.2 | 7.9 | 8.8 | | 7.6 | 8.7 | 0.0 | 7.5 | | 8.5 | 9.2 | 11.6 | 9.5 | | 16.0 | 10.1 | 9.8 | 9.8 | |
| 104 | Low risk | Muranga | Kangema | 2.2 | 7.7 | 10.8 | 12.5 | | 11.6 | 11.6 | 10.8 | 10.2 | | 11.5 | 11.8 | 12.3 | 2.7 | | 13.3 | 14.1 | 14.3 | 12.8 | |
| 105 | Low risk | Muranga | Mathioya | 2.8 | 10.6 | 14.5 | 16.5 | | 12.5 | 15.8 | 14.8 | 14.4 | | 15.4 | 15.6 | 16.3 | 17.1 | | 17.9 | 20.4 | 19.3 | 18.5 | |
| 106 | Low risk | Muranga | Kiharu | 1.4 | 11.1 | 15.4 | 20.4 | | 16.4 | 17.1 | 18.3 | 17.2 | | 17.5 | 17.8 | 18.1 | 18.7 | | 18.8 | 19.8 | 16.0 | 19.4 | |
| 107 | Low risk | Muranga | Kigumo | 1.8 | 11.3 | 14.6 | 15.7 | | 14.0 | 18.0 | 11.4 | 13.8 | | 15.2 | 15.1 | 14.5 | 16.0 | | 16.9 | 18.6 | 18.2 | 17.6 | |
| 108 | Low risk | Muranga | Maragwa | 1.2 | 12.7 | 17.0 | 20.2 | | 17.6 | 16.5 | 15.3 | 18.1 | | 19.0 | 21.3 | 21.0 | 20.7 | | 20.9 | 22.8 | 24.0 | 21.9 | |
| 109 | Low risk | Muranga | Kandara | 1.5 | 8.2 | 12.3 | 14.3 | | 12.7 | 13.8 | 0.0 | 13.7 | | 14.6 | 14.3 | 14.6 | 14.9 | | 15.1 | 16.6 | 16.1 | 16.4 | |
| 110 | Low risk | Muranga | Gatanga | 2.2 | 8.2 | 11.3 | 12.9 | | 11.3 | 11.7 | 10.1 | 11.1 | | 12.2 | 12.5 | 13.1 | 13.3 | | 13.6 | 14.8 | 15.0 | 14.0 | |
| 111 | Low risk | Kiambu | Gatundu South | 5.7 | 21.4 | 28.2 | 33.6 | | 29.6 | 19.0 | 25.6 | 28.3 | | 31.7 | 32.7 | 34.8 | 77.7 | | 50.5 | 38.8 | 38.9 | 38.5 | |
| 112 | Low risk | Kiambu | Gatundu North | 0.6 | 2.7 | 3.7 | 4.3 | | 3.8 | 4.0 | 3.3 | 3.7 | | 4.1 | 4.4 | 4.3 | 3.8 | | 4.6 | 5.0 | 5.2 | 5.0 | |
| 113 | Low risk | Kiambu | Juja | 1.4 | 12.6 | 19.9 | 19.6 | | 17.4 | 18.4 | 10.1 | 17.1 | | 18.9 | 18.5 | 20.0 | 21.4 | | 20.8 | 22.7 | 22.9 | 21.7 | |
| 114 | Low risk | Kiambu | Thika Town | 2.4 | 14.8 | 20.2 | 23.2 | | 19.5 | 22.1 | 17.7 | 20.1 | | 22.5 | 24.5 | 23.6 | 25.7 | | 25.6 | 27.4 | 26.7 | 26.5 | |
| 115 | Low risk | Kiambu | Ruiru | 6.3 | 28.5 | 32.4 | 43.5 | | 39.6 | 43.1 | 34.0 | 42.4 | | 42.3 | 46.2 | 45.3 | 45.8 | | 47.4 | 49.6 | 50.0 | 47.6 | |
| 116 | Low risk | Kiambu | Githunguri | 0.8 | 5.1 | 7.5 | 8.5 | | 7.8 | 8.0 | 6.8 | 8.0 | | 9.1 | 9.0 | 9.1 | 9.1 | | 9.5 | 10.4 | 10.7 | 10.1 | |
| 117 | Low risk | Kiambu | Kiambu Town | 7.4 | 14.1 | 66.1 | 58.6 | | 73.7 | 64.3 | 55.2 | 71.4 | | 64.7 | 66.4 | 69.0 | 79.8 | | 69.2 | 76.5 | 78.7 | 73.8 | |
| 118 | Low risk | Kiambu | Kiambaa | 1.1 | 16.5 | 22.1 | 24.0 | | 21.3 | 22.2 | 20.1 | 21.1 | | 24.4 | 24.1 | 24.5 | 24.8 | | 25.5 | 27.6 | 27.2 | 25.4 | |
| 119 | Low risk | Kiambu | Kabete | 1.2 | 5.2 | 3.1 | 8.6 | | 7.2 | 7.3 | 3.0 | 7.2 | | 7.9 | 8.1 | 17.1 | 8.8 | | 9.1 | 10.3 | 20.6 | 28.0 | |
| 120 | Low risk | Kiambu | Kikuyu | 1.7 | 7.4 | 9.3 | 9.9 | | 8.5 | 9.4 | 7.6 | 8.6 | | 9.8 | 10.1 | 10.3 | 10.5 | | 10.3 | 11.9 | 11.6 | 11.2 | |
| 121 | Low risk | Kiambu | Limuru | 2.0 | 9.7 | 12.7 | 13.7 | | 12.7 | 13.0 | 10.9 | 12.2 | | 13.3 | 14.3 | 14.7 | 15.4 | | 16.4 | 17.8 | 19.0 | 16.8 | |
| 122 | Low risk | Kiambu | Lari | 0.6 | 2.2 | 3.0 | 3.3 | | 3.0 | 2.9 | 2.4 | 2.9 | | 3.1 | 3.3 | 3.4 | 3.6 | | 3.5 | 3.9 | 3.9 | 3.8 | |
| 123 | Seasonal | Turkana | Turkana North | 0.2 | 10.2 | 1.5 | 2.7 | | 1.5 | 1.9 | 1.3 | 1.5 | | 1.6 | 1.4 | 1.6 | 0.9 | | 1.1 | 2.0 | 1.9 | 1.5 | |
| 124 | Seasonal | Turkana | Turkana West | 0.4 | 2.1 | 2.4 | 8.8 | | 17.8 | 3.6 | 2.0 | 2.3 | | 2.4 | 1.2 | 3.3 | 2.8 | | 2.8 | 3.0 | 3.1 | 4.3 | |
| 125 | Seasonal | Turkana | Turkana Central | 1.3 | 5.7 | 6.2 | 1.7 | | 9.6 | 10.6 | 8.7 | 24.1 | | 10.3 | 10.6 | 10.9 | 11.2 | | 11.6 | 10.4 | 12.9 | 20.7 | |
| 126 | Seasonal | Turkana | Loima | 1.1 | 6.0 | 8.2 | 6.7 | | 6.5 | 6.3 | 5.0 | 5.7 | | 6.3 | 6.4 | 2.4 | 2.9 | | 7.0 | 7.5 | 7.2 | 5.9 | |
| 127 | Seasonal | Turkana | Turkana South | 1.4 | 10.2 | 8.8 | 10.1 | | 9.4 | 9.1 | 9.1 | 8.0 | | 9.4 | 9.6 | 5.0 | 4.2 | | 10.3 | 11.6 | 11.5 | 11.1 | |
| 128 | Seasonal | Turkana | Turkana East | 0.8 | 4.1 | 4.2 | 5.1 | | 6.6 | 4.9 | 3.9 | 4.4 | | 5.3 | 5.1 | 5.0 | 3.6 | | 6.6 | 7.3 | 2.2 | 5.5 | |
| 129 | Highland | West Pokot | Kapenguria | 8.8 | 15.2 | 13.2 | 13.1 | | 10.3 | 10.8 | 8.8 | 9.9 | | 11.1 | 11.0 | 11.8 | 11.9 | | 12.2 | 13.4 | 13.5 | 12.5 | |
| 130 | Highland | West Pokot | Sigor | 16.4 | 20.1 | 21.7 | 16.1 | | 15.7 | 16.1 | 11.0 | 15.1 | | 15.4 | 17.2 | 15.6 | 16.3 | | 16.8 | 18.6 | 17.8 | 17.7 | |
| 131 | Highland | West Pokot | Kacheliba | 2.3 | 10.6 | 17.7 | 9.1 | | 11.7 | 13.4 | 5.4 | 10.9 | | 13.5 | 14.1 | 14.8 | 18.9 | | 19.4 | 18.8 | 3.6 | 3.3 | |
| 132 | Highland | West Pokot | Pokot South | 0.4 | 15.0 | 2.1 | 2.6 | | 2.4 | 1.9 | 1.7 | 2.0 | | 2.1 | 2.0 | 2.0 | 2.3 | | 1.7 | 1.8 | 2.5 | 2.2 | |
| 133 | Seasonal | Samburu | Samburu Central | 2.3 | 9.4 | 13.1 | 15.9 | | 13.9 | 13.5 | 12.6 | 12.9 | | 16.1 | 15.0 | 7.8 | 3.6 | | 13.6 | 15.8 | 16.1 | 13.4 | |
| 134 | Seasonal | Samburu | Samburu North | 0.9 | 1.9 | 4.5 | 4.0 | | 4.5 | 4.1 | 4.7 | 15.7 | | 8.7 | 19.8 | 5.2 | 1.8 | | 8.5 | 4.6 | 4.4 | 7.2 | |
| 135 | Seasonal | Samburu | Samburu East | 0.7 | 2.8 | 2.2 | 4.7 | | 4.5 | 4.4 | 5.4 | 5.0 | | 5.5 | 5.0 | 4.8 | 3.1 | | 5.2 | 6.0 | 7.0 | 5.2 | |
| 136 | Highland | Trans Nzoia | Kwanza | 0.7 | 5.1 | 8.0 | 7.7 | | 7.0 | 7.0 | 5.1 | 6.6 | | 6.8 | 7.9 | 11.9 | 10.9 | | 9.0 | 8.9 | 8.8 | 7.8 | |
| 137 | Highland | Trans Nzoia | Endebess | 0.8 | 2.7 | 3.1 | 3.7 | | 5.0 | 6.2 | 4.7 | 5.7 | | 6.4 | 7.9 | 23.9 | 10.7 | | 7.8 | 8.0 | 7.9 | 7.6 | |
| 138 | Highland | Trans Nzoia | Saboti | 0.5 | 2.4 | 1.6 | 4.6 | | 3.4 | 3.8 | 3.6 | 2.7 | | 3.0 | 3.1 | 3.9 | 4.0 | | 3.9 | 4.7 | 4.8 | 5.6 | |
| 139 | Highland | Trans Nzoia | Kiminini | 5.8 | 11.6 | 15.4 | 17.1 | | 15.3 | 16.1 | 15.8 | 14.4 | | 15.8 | 16.8 | 17.8 | 18.3 | | 20.1 | 20.2 | 20.0 | 18.7 | |
| 140 | Highland | Trans Nzoia | Cherangany | 0.9 | 1.2 | 5.7 | 6.6 | | 5.8 | 6.0 | 5.7 | 5.5 | | 5.5 | 5.3 | 6.7 | 6.7 | | 6.9 | 7.8 | 7.4 | 7.2 | |
| 141 | Highland | Uasin Gishu | Soy | 2.3 | 15.3 | 16.3 | 18.0 | | 16.9 | 17.4 | 14.5 | 15.8 | | 17.7 | 18.1 | 19.4 | 19.6 | | 19.6 | 21.4 | 21.4 | 20.7 | |
| 142 | Highland | Uasin Gishu | Turbo | 6.4 | 20.3 | 8.4 | 8.8 | | 8.3 | 8.8 | 7.2 | 8.1 | | 8.9 | 9.4 | 9.4 | 10.0 | | 10.6 | 11.2 | 11.4 | 10.7 | |
| 143 | Highland | Uasin Gishu | Moiben | 4.2 | 22.3 | 27.3 | 27.0 | | 23.7 | 26.8 | 21.1 | 23.6 | | 25.5 | 26.9 | 26.8 | 27.8 | | 27.8 | 30.6 | 29.5 | 28.8 | |
| 144 | Highland | Uasin Gishu | Ainabkoi | 2.1 | 10.5 | 14.2 | 16.4 | | 15.0 | 15.5 | 12.3 | 14.2 | | 15.6 | 15.5 | 16.6 | 16.9 | | 17.3 | 19.1 | 19.0 | 18.0 | |
| 145 | Highland | Uasin Gishu | Kapseret | 2.8 | 15.1 | 18.0 | 15.2 | | 18.5 | 19.4 | 16.2 | 19.3 | | 18.9 | 20.3 | 20.8 | 21.3 | | 21.7 | 22.8 | 24.0 | 23.1 | |
| 146 | Highland | Uasin Gishu | Kesses | 2.3 | 7.8 | 10.7 | 11.8 | | 10.5 | 11.3 | 9.2 | 10.1 | | 10.6 | 11.8 | 12.1 | 12.5 | | 12.8 | 14.0 | 14.0 | 14.5 | |
| 147 | Seasonal | Elgeyo-Marakwet | Marakwet East | 0.8 | 1.8 | 4.9 | 5.6 | | 4.9 | 4.5 | 2.5 | 4.8 | | 5.3 | 5.6 | 5.9 | 6.2 | | 5.9 | 6.8 | 7.3 | 6.7 | |
| 148 | Seasonal | Elgeyo-Marakwet | Marakwet West | 1.5 | 5.7 | 6.3 | 8.4 | | 7.6 | 8.4 | 5.7 | 4.9 | | 8.1 | 8.4 | 10.0 | 9.3 | | 9.1 | 10.6 | 11.1 | 9.8 | |
| 149 | Seasonal | Elgeyo-Marakwet | Keiyo North | 2.1 | 12.8 | 17.0 | 19.6 | | 17.4 | 18.3 | 12.7 | 16.9 | | 18.7 | 19.7 | 20.3 | 21.1 | | 21.9 | 34.2 | 26.8 | 24.3 | |
| 150 | Seasonal | Elgeyo-Marakwet | Keiyo South | 3.8 | 14.3 | 19.1 | 21.8 | | 19.3 | 21.4 | 17.6 | 18.8 | | 20.5 | 21.4 | 22.0 | 22.8 | | 23.3 | 25.6 | 27.3 | 24.4 | |
| 151 | Highland | Nandi | Tinderet | 3.1 | 6.9 | 20.5 | 20.9 | | 19.7 | 20.1 | 3.5 | 18.1 | | 20.8 | 22.1 | 22.7 | 24.7 | | 23.7 | 25.8 | 25.6 | 22.1 | |
| 152 | Highland | Nandi | Aldai | 1.4 | 6.3 | 9.4 | 10.5 | | 10.2 | 10.5 | 2.3 | 5.9 | | 7.8 | 9.5 | 10.7 | 11.0 | | 11.0 | 13.1 | 12.5 | 11.8 | |
| 153 | Highland | Nandi | Nandi Hills | 2.0 | 11.6 | 32.9 | 34.3 | | 23.1 | 16.6 | 4.9 | 12.9 | | 24.9 | 19.9 | 16.1 | 13.6 | | 18.7 | 20.6 | 21.1 | 12.2 | |
| 154 | Highland | Nandi | Chesumei | 4.2 | 15.0 | 20.9 | 22.5 | | 20.0 | 21.5 | 10.9 | 17.8 | | 12.3 | 24.3 | 24.0 | 36.2 | | 29.3 | 28.1 | 28.6 | 26.5 | |
| 155 | Highland | Nandi | Emgwen | 2.0 | 2.9 | 15.4 | 17.5 | | 15.3 | 16.3 | 11.9 | 15.2 | | 16.2 | 17.0 | 18.2 | 18.5 | | 23.6 | 25.3 | 20.8 | 19.6 | |
| 156 | Highland | Nandi | Mosop | 2.2 | 10.7 | 15.0 | 16.9 | | 15.1 | 15.4 | 13.2 | 14.2 | | 15.4 | 16.4 | 16.7 | 17.0 | | 15.8 | 19.1 | 19.1 | 18.0 | |
| 157 | Highland | Baringo | Tiaty | 7.3 | 8.4 | 11.3 | 12.6 | | 11.6 | 12.2 | 9.9 | 10.7 | | 12.4 | 12.5 | 13.0 | 12.9 | | 15.8 | 15.3 | 14.9 | 14.5 | |
| 158 | Highland | Baringo | Baringo North | 27.0 | 17.5 | 25.1 | 28.1 | | 25.3 | 26.3 | 21.9 | 24.3 | | 27.0 | 28.5 | 29.2 | 29.7 | | 31.5 | 34.0 | 33.9 | 32.2 | |
| 159 | Highland | Baringo | Baringo Central | 2.0 | 12.8 | 14.0 | 15.8 | | 14.4 | 14.8 | 12.4 | 13.5 | | 15.2 | 15.6 | 16.2 | 15.9 | | 16.9 | 18.4 | 18.6 | 17.0 | |
| 160 | Highland | Baringo | Baringo South | 4.9 | 10.0 | 12.6 | 10.4 | | 11.9 | 13.1 | 11.9 | 12.4 | | 14.0 | 14.2 | 14.9 | 15.7 | | 17.2 | 17.5 | 18.1 | 17.1 | |
| 161 | Highland | Baringo | Mogotio | 1.4 | 9.2 | 15.7 | 16.5 | | 16.2 | 17.0 | 13.9 | 15.7 | | 17.9 | 19.1 | 20.7 | 20.2 | | 21.1 | 21.9 | 22.1 | 21.0 | |
| 162 | Highland | Baringo | Eldama Ravine | 1.0 | 7.9 | 10.9 | 11.1 | | 9.8 | 9.8 | 10.7 | 9.6 | | 10.6 | 11.4 | 11.2 | 11.2 | | 11.1 | 12.6 | 12.5 | 11.8 | |
| 163 | Low risk | Laikipia | Laikipia West | 1.1 | 11.0 | 15.6 | 16.9 | | 15.0 | 16.3 | 13.2 | 14.5 | | 16.0 | 16.9 | 17.3 | 18.3 | | 18.2 | 19.9 | 20.1 | 19.6 | |
| 164 | Low risk | Laikipia | Laikipia East | 1.4 | 2.4 | 8.7 | 9.8 | | 8.7 | 9.5 | 8.2 | 10.0 | | 10.0 | 9.8 | 10.9 | 10.5 | | 10.7 | 11.6 | 11.8 | 11.1 | |
| 165 | Low risk | Laikipia | Laikipia North | 1.2 | 4.6 | 3.1 | 7.3 | | 4.4 | 6.1 | 5.6 | 6.0 | | 6.4 | 7.2 | 7.7 | 7.7 | | 7.9 | 9.1 | 9.0 | 8.5 | |
| 166 | Low risk | Nakuru | Molo | 0.7 | 5.3 | 6.9 | 6.9 | | 7.1 | 7.5 | 5.5 | 6.8 | | 7.5 | 7.5 | 8.1 | 8.5 | | 27.0 | 9.4 | 14.0 | 19.4 | |
| 167 | Low risk | Nakuru | Njoro | 4.7 | 17.8 | 24.1 | 27.3 | | 21.0 | 22.3 | 20.9 | 22.7 | | 25.6 | 27.2 | 27.7 | 28.7 | | 31.4 | 35.1 | 36.5 | 46.8 | |
| 168 | Low risk | Nakuru | Naivasha | 1.5 | 8.4 | 7.3 | 8.6 | | 7.6 | 8.0 | 7.7 | 8.9 | | 8.1 | 8.1 | 8.3 | 9.0 | | 9.1 | 9.7 | 9.9 | 9.4 | |
| 169 | Low risk | Nakuru | Gilgil | 1.1 | 4.1 | 5.7 | 3.8 | | 4.6 | 6.1 | 5.1 | 5.5 | | 6.1 | 6.3 | 6.4 | 6.6 | | 6.9 | 7.5 | 7.6 | 7.4 | |
| 170 | Low risk | Nakuru | Kuresoi South | 1.6 | 4.3 | 8.3 | 10.0 | | 8.6 | 9.2 | 7.0 | 8.9 | | 9.2 | 9.4 | 9.7 | 9.8 | | 10.4 | 10.9 | 11.2 | 10.7 | |
| 171 | Low risk | Nakuru | Kuresoi North | 0.4 | 1.8 | 1.3 | 1.4 | | 2.0 | 2.0 | 1.7 | 2.0 | | 2.2 | 2.3 | 2.3 | 2.5 | | 9.5 | 5.6 | 5.5 | 7.8 | |
| 172 | Low risk | Nakuru | Subukia | 13.9 | 7.8 | 11.8 | 13.0 | | 11.7 | 12.6 | 10.3 | 11.1 | | 12.2 | 12.5 | 13.1 | 13.4 | | 13.4 | 16.0 | 15.3 | 14.2 | |
| 173 | Low risk | Nakuru | Rongai | 2.8 | 10.3 | 14.0 | 16.2 | | 14.1 | 13.3 | 12.3 | 13.7 | | 15.5 | 15.9 | 16.7 | 16.8 | | 18.5 | 18.7 | 18.7 | 17.8 | |
| 174 | Low risk | Nakuru | Bahati | 0.9 | 2.2 | 3.9 | 7.5 | | 6.5 | 7.2 | 5.9 | 6.2 | | 6.9 | 7.5 | 7.8 | 7.8 | | 8.0 | 8.9 | 8.8 | 8.5 | |
| 175 | Low risk | Nakuru | Nakuru West | 1.4 | 16.9 | 23.4 | 25.6 | | 22.9 | 28.2 | 20.5 | 23.1 | | 26.8 | 27.0 | 27.3 | 28.3 | | 23.8 | 31.5 | 32.4 | 30.2 | |
| 176 | Low risk | Nakuru | Nakuru East | 2.1 | 12.2 | 18.5 | 17.9 | | 15.5 | 16.0 | 13.2 | 14.9 | | 16.0 | 16.8 | 17.1 | 18.0 | | 18.5 | 18.8 | 19.8 | 19.0 | |
| 177 | Highland | Narok | Kilgoris | 3.9 | 11.2 | 15.1 | 14.7 | | 14.6 | 16.2 | 12.8 | 14.9 | | 16.1 | 16.9 | 17.6 | 18.1 | | 18.3 | 21.2 | 20.4 | 21.6 | |
| 178 | Highland | Narok | Emurua Dikirr | 5.5 | 4.8 | 6.9 | 7.3 | | 4.2 | 2.2 | 2.6 | 1.4 | | 1.8 | 1.6 | 7.4 | 7.6 | | 7.9 | 8.6 | 21.2 | 19.2 | |
| 182 | Highland | Narok | Narok West | 0.6 | 2.9 | 3.6 | 4.0 | | 3.5 | 4.1 | 3.2 | 3.7 | | 2.8 | 4.1 | 4.5 | 4.1 | | 1.8 | 6.3 | 7.2 | 9.8 | |
| 179 | Highland | Narok | Narok North | 1.3 | 12.4 | 16.7 | 19.1 | | 6.3 | 18.2 | 17.8 | 19.8 | | 20.5 | 19.3 | 20.6 | 32.8 | | 20.2 | 24.3 | 22.7 | 21.9 | |
| 180 | Highland | Narok | Narok East | 0.5 | 1.6 | 2.9 | 2.7 | | 2.7 | 3.1 | 2.5 | 2.3 | | 3.7 | 4.3 | 2.9 | 3.5 | | 2.6 | 3.1 | 3.7 | 5.6 | |
| 181 | Highland | Narok | Narok South | 0.6 | 1.7 | 2.1 | 6.8 | | 5.7 | 6.0 | 4.0 | 3.8 | | 6.3 | 6.4 | 6.9 | 6.9 | | 7.6 | 8.8 | 12.3 | 9.6 | |
| 183 | Seasonal | Kajiado | Kajiado North | 9.9 | 12.0 | 15.0 | 17.0 | | 15.3 | 15.9 | 26.7 | 14.5 | | 16.1 | 16.6 | 16.4 | 16.5 | | 18.0 | 20.7 | 20.2 | 19.6 | |
| 184 | Seasonal | Kajiado | Kajiado Central | 4.9 | 26.6 | 25.5 | 29.3 | | 25.9 | 27.6 | 28.3 | 24.6 | | 27.7 | 27.5 | 28.6 | 28.5 | | 29.9 | 33.2 | 32.9 | 32.0 | |
| 185 | Seasonal | Kajiado | Kajiado West | 6.1 | 13.0 | 17.0 | 21.0 | | 17.4 | 18.8 | 13.8 | 16.2 | | 18.8 | 18.9 | 18.0 | 19.8 | | 20.4 | 22.0 | 21.9 | 21.5 | |
| 186 | Seasonal | Kajiado | Kajiado East | 3.4 | 15.3 | 20.7 | 23.9 | | 20.8 | 22.0 | 18.8 | 20.7 | | 22.6 | 22.5 | 24.2 | 24.8 | | 25.4 | 28.3 | 28.0 | 27.8 | |
| 187 | Seasonal | Kajiado | Kajiado South | 4.0 | 18.8 | 25.7 | 29.9 | | 25.7 | 27.1 | 22.9 | 24.6 | | 27.4 | 28.9 | 29.4 | 30.2 | | 31.2 | 33.3 | 33.8 | 32.3 | |
| 188 | Highland | Kericho | Kipkelion East | 1.9 | 9.1 | 12.7 | 12.8 | | 13.0 | 13.6 | 0.0 | 12.3 | | 16.0 | 14.4 | 14.8 | 15.4 | | 15.5 | 17.4 | 16.7 | 16.3 | |
| 189 | Highland | Kericho | Kipkelion West | 4.2 | 17.2 | 21.5 | 23.3 | | 20.6 | 23.7 | 21.8 | 20.1 | | 22.0 | 23.1 | 22.7 | 24.2 | | 24.2 | 27.3 | 27.3 | 26.3 | |
| 190 | Highland | Kericho | Ainamoi | 6.0 | 27.6 | 39.5 | 43.4 | | 38.5 | 40.4 | 32.0 | 36.4 | | 42.2 | 44.6 | 42.9 | 43.5 | | 45.3 | 49.6 | 50.7 | 47.0 | |
| 191 | Highland | Kericho | Bureti | 4.3 | 19.9 | 28.0 | 29.4 | | 27.0 | 28.4 | 23.7 | 25.5 | | 28.5 | 29.7 | 30.1 | 31.7 | | 32.9 | 35.1 | 37.2 | 35.2 | |
| 192 | Highland | Kericho | Belgut | 2.3 | 9.9 | 13.7 | 15.2 | | 13.8 | 14.4 | 13.8 | 13.6 | | 14.9 | 15.3 | 16.0 | 16.2 | | 16.5 | 18.1 | 18.1 | 17.4 | |
| 193 | Highland | Kericho | Sigowet/Soin | 4.4 | 19.4 | 27.1 | 30.4 | | 27.0 | 28.2 | 23.9 | 25.8 | | 27.9 | 29.1 | 30.8 | 31.4 | | 32.3 | 35.1 | 35.6 | 34.0 | |
| 194 | Highland | Bomet | Sotik | 2.3 | 10.9 | 15.5 | 16.6 | | 14.6 | 15.5 | 12.8 | 14.2 | | 15.3 | 16.3 | 17.0 | 17.1 | | 16.7 | 19.6 | 20.6 | 22.8 | |
| 195 | Highland | Bomet | Chepalungu | 9.6 | 17.6 | 21.7 | 24.1 | | 21.9 | 22.6 | 18.7 | 23.9 | | 23.0 | 23.4 | 24.9 | 18.4 | | 26.3 | 28.6 | 28.7 | 26.3 | |
| 196 | Highland | Bomet | Bomet East | 1.1 | 9.2 | 14.5 | 13.5 | | 13.9 | 15.5 | 12.4 | 13.9 | | 15.3 | 16.4 | 16.8 | 19.5 | | 18.8 | 18.6 | 27.6 | 23.1 | |
| 197 | Highland | Bomet | Bomet Central | 3.3 | 14.1 | 19.2 | 21.4 | | 18.9 | 19.6 | 16.6 | 18.7 | | 20.2 | 21.3 | 21.7 | 20.7 | | 24.3 | 33.4 | 33.8 | 45.3 | |
| 198 | Highland | Bomet | Konoin | 1.8 | 9.9 | 13.4 | 17.7 | | 15.8 | 16.0 | 13.5 | 14.8 | | 17.5 | 17.1 | 24.0 | 36.3 | | 21.7 | 23.6 | 21.0 | 34.7 | |
| 199 | Lake endemic | Kakamega | Lugari | 0.7 | 3.1 | 2.9 | 4.7 | | 4.2 | 4.5 | 3.5 | 4.1 | | 4.4 | 4.7 | 4.9 | 5.8 | | 5.4 | 5.5 | 5.6 | 5.2 | |
| 200 | Lake endemic | Kakamega | Likuyani | 2.0 | 9.4 | 12.7 | 12.0 | | 9.4 | 11.2 | 9.1 | 9.9 | | 11.7 | 11.7 | 12.4 | 12.4 | | 13.1 | 14.2 | 14.1 | 13.4 | |
| 201 | Lake endemic | Kakamega | Malava | 0.5 | 4.9 | 10.4 | 11.5 | | 10.3 | 10.8 | 9.7 | 10.0 | | 7.9 | 11.5 | 12.1 | 12.5 | | 11.1 | 12.0 | 14.7 | 12.6 | |
| 202 | Lake endemic | Kakamega | Lurambi | 1.4 | 5.7 | 9.7 | 10.4 | | 10.0 | 11.2 | 9.1 | 10.5 | | 11.3 | 11.2 | 11.3 | 11.4 | | 12.4 | 13.0 | 13.1 | 12.5 | |
| 203 | Lake endemic | Kakamega | Navakholo | 1.0 | 6.6 | 9.3 | 10.2 | | 9.4 | 9.8 | 7.7 | 9.8 | | 9.0 | 10.3 | 10.7 | 11.2 | | 11.7 | 12.1 | 12.4 | 11.4 | |
| 204 | Lake endemic | Kakamega | Mumias West | 1.3 | 1.6 | 9.8 | 10.9 | | 9.3 | 10.8 | 8.7 | 9.6 | | 10.4 | 11.0 | 11.0 | 10.5 | | 12.6 | 13.1 | 13.1 | 12.8 | |
| 205 | Lake endemic | Kakamega | Mumias East | 8.3 | 8.8 | 4.9 | 5.3 | | 5.0 | 5.7 | 3.9 | 1.7 | | 4.9 | 2.4 | 5.1 | 5.2 | | 6.3 | 6.0 | 7.0 | 4.6 | |
| 206 | Lake endemic | Kakamega | Matungu | 1.6 | 8.0 | 10.9 | 12.6 | | 11.2 | 11.7 | 11.7 | 10.3 | | 11.0 | 12.8 | 13.1 | 12.9 | | 13.3 | 14.5 | 17.3 | 13.8 | |
| 207 | Lake endemic | Kakamega | Butere | 2.7 | 13.6 | 19.0 | 24.1 | | 21.6 | 22.2 | 17.8 | 20.5 | | 22.6 | 24.0 | 25.4 | 24.8 | | 25.9 | 27.8 | 29.6 | 26.6 | |
| 208 | Lake endemic | Kakamega | Khwisero | 1.2 | 1.7 | 5.7 | 9.1 | | 8.2 | 10.0 | 8.0 | 9.2 | | 9.0 | 11.0 | 12.1 | 11.1 | | 14.7 | 14.1 | 13.5 | 12.2 | |
| 209 | Lake endemic | Kakamega | Shinyalu | 0.7 | 1.6 | 1.7 | 6.8 | | 6.1 | 6.5 | 5.8 | 6.0 | | 6.0 | 6.9 | 7.0 | 6.9 | | 7.2 | 8.0 | 8.1 | 7.9 | |
| 210 | Lake endemic | Kakamega | Ikolomani | 1.2 | 6.9 | 9.5 | 10.9 | | 8.7 | 10.1 | 25.6 | 10.8 | | 10.3 | 11.1 | 11.2 | 11.2 | | 11.5 | 12.6 | 12.7 | 11.6 | |
| 211 | Lake endemic | Vihiga | Vihiga | 1.8 | 2.8 | 8.4 | 10.3 | | 9.1 | 10.3 | 9.4 | 8.5 | | 9.1 | 9.9 | 10.6 | 10.6 | | 10.7 | 11.6 | 12.0 | 10.9 | |
| 212 | Lake endemic | Vihiga | Sabatia | 1.9 | 9.8 | 13.7 | 13.0 | | 13.2 | 14.1 | 7.9 | 4.8 | | 14.7 | 18.7 | 18.0 | 16.2 | | 16.1 | 18.9 | 18.5 | 17.1 | |
| 213 | Lake endemic | Vihiga | Hamisi | 1.5 | 9.6 | 17.6 | 21.0 | | 14.1 | 14.7 | 13.9 | 13.2 | | 14.5 | 15.2 | 15.3 | 16.1 | | 16.1 | 17.1 | 16.5 | 16.7 | |
| 214 | Lake endemic | Vihiga | Luanda | 2.5 | 12.3 | 16.8 | 19.0 | | 16.0 | 18.0 | 27.2 | 16.3 | | 18.1 | 18.6 | 19.1 | 19.3 | | 19.9 | 21.3 | 22.5 | 20.9 | |
| 215 | Lake endemic | Vihiga | Emuhaya | 2.8 | 13.3 | 18.1 | 24.9 | | 17.9 | 27.6 | 15.0 | 17.7 | | 18.9 | 21.1 | 20.8 | 21.2 | | 20.6 | 21.6 | 23.3 | 21.7 | |
| 216 | Lake endemic | Bungoma | Mt. Elgon | 0.4 | 0.4 | 2.0 | 4.1 | | 3.7 | 4.2 | 3.1 | 3.6 | | 4.0 | 3.9 | 4.2 | 4.3 | | 4.3 | 4.7 | 4.8 | 4.5 | |
| 217 | Lake endemic | Bungoma | Sirisia | 1.7 | 8.4 | 3.7 | 9.3 | | 7.6 | 9.1 | 7.8 | 8.6 | | 3.6 | 9.4 | 10.1 | 11.0 | | 10.5 | 11.9 | 11.7 | 11.1 | |
| 218 | Lake endemic | Bungoma | Kabuchai | 1.5 | 9.3 | 14.4 | 15.9 | | 12.7 | 14.2 | 11.5 | 12.8 | | 14.3 | 14.2 | 15.5 | 15.5 | | 16.1 | 16.7 | 17.5 | 16.9 | |
| 219 | Lake endemic | Bungoma | Bumula | 1.7 | 4.1 | 16.6 | 17.8 | | 17.2 | 18.4 | 11.1 | 17.3 | | 19.2 | 19.6 | 20.0 | 20.5 | | 21.3 | 23.5 | 23.2 | 23.4 | |
| 220 | Lake endemic | Bungoma | Kanduyi | 2.9 | 13.9 | 20.4 | 21.9 | | 19.6 | 20.0 | 16.7 | 18.4 | | 20.5 | 21.2 | 22.0 | 22.3 | | 22.9 | 25.3 | 25.4 | 24.1 | |
| 221 | Lake endemic | Bungoma | Webuye East | 2.1 | 7.5 | 10.1 | 11.3 | | 8.9 | 5.5 | 7.0 | 10.3 | | 11.5 | 12.3 | 12.3 | 12.4 | | 12.8 | 13.9 | 15.0 | 15.6 | |
| 222 | Lake endemic | Bungoma | Webuye West | 0.8 | 1.5 | 9.0 | 10.9 | | 8.3 | 9.7 | 3.0 | 8.6 | | 9.6 | 10.0 | 10.3 | 10.6 | | 11.2 | 12.0 | 12.1 | 17.8 | |
| 223 | Lake endemic | Bungoma | Kimilili | 3.6 | 5.4 | 10.4 | 21.6 | | 18.9 | 20.1 | 13.1 | 16.9 | | 20.1 | 24.5 | 21.9 | 23.2 | | 23.0 | 25.7 | 25.6 | 24.0 | |
| 224 | Lake endemic | Bungoma | Tongaren | 1.6 | 7.3 | 9.7 | 10.8 | | 9.9 | 11.2 | 7.7 | 9.2 | | 10.3 | 10.9 | 11.3 | 11.6 | | 11.7 | 13.2 | 70.6 | 11.7 | |
| 225 | Lake endemic | Busia | Teso North | 4.3 | 12.5 | 16.9 | 16.9 | | 16.4 | 15.9 | 0.0 | 16.2 | | 18.4 | 18.9 | 19.7 | 20.3 | | 20.8 | 22.5 | 22.3 | 21.7 | |
| 226 | Lake endemic | Busia | Teso South | 2.5 | 11.9 | 15.2 | 17.2 | | 15.6 | 14.9 | 9.2 | 15.3 | | 17.1 | 18.8 | 19.6 | 19.1 | | 21.6 | 22.1 | 22.0 | 20.3 | |
| 227 | Lake endemic | Busia | Nambale | 5.7 | 12.1 | 5.3 | 6.0 | | 8.6 | 19.7 | 8.8 | 16.8 | | 20.5 | 20.9 | 22.9 | 23.2 | | 23.4 | 25.9 | 25.9 | 24.1 | |
| 228 | Lake endemic | Busia | Matayos | 3.5 | 13.1 | 21.6 | 22.5 | | 20.5 | 22.8 | 15.3 | 21.8 | | 23.8 | 25.4 | 26.0 | 25.6 | | 26.4 | 28.6 | 29.0 | 28.5 | |
| 229 | Lake endemic | Busia | Butula | 2.5 | 11.1 | 14.9 | 17.6 | | 15.3 | 15.8 | 9.9 | 14.6 | | 16.5 | 17.1 | 17.7 | 18.0 | | 18.9 | 21.2 | 20.7 | 19.2 | |
| 230 | Lake endemic | Busia | Funyula | 6.9 | 17.9 | 27.1 | 30.3 | | 26.4 | 28.0 | 21.8 | 24.3 | | 28.4 | 29.8 | 31.4 | 32.2 | | 31.8 | 35.3 | 35.5 | 33.6 | |
| 231 | Lake endemic | Busia | Budalangi | 13.8 | 29.2 | 39.7 | 44.4 | | 39.4 | 42.4 | 34.8 | 37.7 | | 42.1 | 44.0 | 46.1 | 46.7 | | 47.5 | 49.0 | 52.0 | 48.6 | |
| 232 | Lake endemic | Siaya | Ugenya | 2.1 | 11.2 | 15.6 | 17.1 | | 13.5 | 16.2 | 7.7 | 15.1 | | 18.5 | 18.3 | 18.2 | 18.4 | | 19.4 | 20.2 | 23.7 | 19.6 | |
| 233 | Lake endemic | Siaya | Ugunja | 3.9 | 26.5 | 36.7 | 40.2 | | 35.2 | 38.0 | 31.6 | 34.2 | | 38.8 | 39.1 | 40.7 | 41.7 | | 44.4 | 45.7 | 47.9 | 45.0 | |
| 234 | Lake endemic | Siaya | Alego Usonga | 3.8 | 18.1 | 25.1 | 27.7 | | 24.2 | 25.7 | 21.4 | 23.4 | | 25.3 | 26.6 | 28.0 | 28.2 | | 28.2 | 30.4 | 31.9 | 30.4 | |
| 235 | Lake endemic | Siaya | Gem | 3.1 | 24.5 | 26.4 | 29.5 | | 26.1 | 29.8 | 22.8 | 25.5 | | 28.1 | 28.7 | 28.8 | 30.5 | | 30.3 | 31.4 | 32.7 | 31.4 | |
| 236 | Lake endemic | Siaya | Bondo | 7.5 | 35.7 | 44.6 | 49.3 | | 44.5 | 47.0 | 39.9 | 43.1 | | 47.4 | 48.4 | 50.5 | 51.4 | | 52.3 | 49.9 | 56.7 | 51.2 | |
| 237 | Lake endemic | Siaya | Rarieda | 4.9 | 25.4 | 33.0 | 37.1 | | 33.4 | 36.3 | 29.8 | 32.1 | | 35.7 | 36.1 | 37.9 | 38.6 | | 39.4 | 42.0 | 43.6 | 39.7 | |
| 238 | Lake endemic | Kisumu | Kisumu East | 5.1 | 24.8 | 33.8 | 37.3 | | 34.6 | 36.7 | 44.1 | 32.5 | | 36.5 | 37.5 | 38.7 | 39.2 | | 40.5 | 43.5 | 44.1 | 41.9 | |
| 239 | Lake endemic | Kisumu | Kisumu West | 5.6 | 24.4 | 34.6 | 37.8 | | 32.6 | 35.7 | 29.0 | 32.0 | | 37.6 | 37.7 | 38.4 | 38.3 | | 39.6 | 41.4 | 42.2 | 28.5 | |
| 240 | Lake endemic | Kisumu | Kisumu Central | 7.5 | 52.2 | 67.9 | 74.3 | | 67.8 | 70.6 | 58.8 | 65.1 | | 71.9 | 73.8 | 77.0 | 79.9 | | 80.9 | 87.2 | 87.7 | 82.2 | |
| 241 | Lake endemic | Kisumu | Seme | 4.5 | 33.5 | 44.2 | 47.4 | | 42.2 | 44.2 | 36.5 | 40.5 | | 45.3 | 48.8 | 48.3 | 48.9 | | 49.2 | 52.4 | 53.7 | 39.5 | |
| 242 | Lake endemic | Kisumu | Nyando | 38.3 | 59.5 | 63.7 | 65.4 | | 57.7 | 61.1 | 50.1 | 56.5 | | 61.1 | 63.0 | 65.3 | 64.7 | | 67.4 | 71.9 | 72.8 | 64.5 | |
| 243 | Lake endemic | Kisumu | Muhoroni | 4.2 | 22.8 | 26.0 | 26.9 | | 26.9 | 28.5 | 23.1 | 26.1 | | 28.1 | 30.0 | 30.0 | 31.0 | | 32.0 | 35.8 | 34.9 | 32.0 | |
| 244 | Lake endemic | Kisumu | Nyakach | 7.3 | 33.8 | 44.8 | 49.9 | | 45.2 | 47.8 | 37.8 | 43.3 | | 47.5 | 49.2 | 51.4 | 52.7 | | 52.5 | 55.5 | 56.2 | 54.5 | |
| 245 | Lake endemic | Homa Bay | Kasipul | 0.9 | 5.0 | 2.8 | 11.6 | | 8.7 | 1.7 | 8.4 | 7.7 | | 10.1 | 10.8 | 10.7 | 11.2 | | 11.3 | 12.5 | 12.6 | 12.2 | |
| 246 | Lake endemic | Homa Bay | Kabondo Kasipul | 2.6 | 9.2 | 12.0 | 13.6 | | 11.9 | 12.9 | 10.4 | 11.7 | | 12.8 | 13.3 | 13.6 | 14.1 | | 14.4 | 15.9 | 15.9 | 15.3 | |
| 247 | Lake endemic | Homa Bay | Karachuonyo | 6.5 | 9.3 | 20.1 | 16.6 | | 13.5 | 13.7 | 11.4 | 12.9 | | 14.0 | 12.0 | 14.8 | 14.5 | | 14.7 | 16.9 | 16.7 | 16.5 | |
| 248 | Lake endemic | Homa Bay | Rangwe | 2.6 | 12.8 | 34.1 | 20.1 | | 16.5 | 16.2 | 12.6 | 14.3 | | 16.4 | 13.1 | 16.9 | 7.5 | | 15.8 | 19.2 | 18.8 | 17.4 | |
| 249 | Lake endemic | Homa Bay | Homa Bay | 3.1 | 5.9 | 26.7 | 13.4 | | 13.4 | 11.9 | 9.7 | 30.2 | | 13.1 | 11.8 | 13.0 | 12.7 | | 12.7 | 14.1 | 12.9 | 13.7 | |
| 250 | Lake endemic | Homa Bay | Ndhiwa | 1.1 | 4.9 | 8.3 | 9.1 | | 8.0 | 8.2 | 6.7 | 7.3 | | 1.7 | 2.9 | 8.9 | 9.4 | | 9.3 | 10.5 | 10.5 | 9.9 | |
| 251 | Lake endemic | Homa Bay | Suba North | 3.1 | 30.3 | 20.5 | 21.5 | | 21.9 | 19.6 | 16.8 | 18.2 | | 19.7 | 21.1 | 21.3 | 21.6 | | 22.7 | 25.1 | 25.3 | 23.5 | |
| 252 | Lake endemic | Homa Bay | Suba South | 1.3 | 6.2 | 8.1 | 9.0 | | 8.2 | 8.8 | 6.8 | 8.0 | | 9.0 | 8.8 | 9.3 | 5.2 | | 7.6 | 11.0 | 11.1 | 10.2 | |
| 253 | Lake endemic | Migori | Rongo | 2.4 | 11.2 | 16.3 | 18.6 | | 16.3 | 16.8 | 13.6 | 15.3 | | 17.1 | 17.9 | 18.7 | 19.4 | | 21.2 | 21.2 | 21.1 | 19.4 | |
| 254 | Lake endemic | Migori | Awendo | 1.0 | 1.8 | 5.7 | 8.1 | | 6.2 | 2.1 | 18.3 | 6.1 | | 6.0 | 7.9 | 7.4 | 7.7 | | 7.6 | 8.7 | 8.0 | 7.1 | |
| 255 | Lake endemic | Migori | Suna East | 8.1 | 19.1 | 29.3 | 16.0 | | 13.5 | 14.0 | 15.8 | 13.1 | | 13.9 | 15.0 | 16.0 | 15.7 | | 15.7 | 18.3 | 18.1 | 17.1 | |
| 256 | Lake endemic | Migori | Suna West | 1.8 | 11.4 | 29.7 | 12.4 | | 11.5 | 14.5 | 13.9 | 10.5 | | 8.1 | 6.3 | 11.0 | 5.7 | | 17.5 | 18.1 | 14.6 | 6.0 | |
| 257 | Lake endemic | Migori | Uriri | 1.6 | 12.1 | 9.1 | 9.9 | | 8.9 | 11.8 | 8.1 | 8.2 | | 5.3 | 9.9 | 10.2 | 10.1 | | 10.1 | 4.2 | 7.6 | 10.0 | |
| 258 | Lake endemic | Migori | Nyatike | 0.8 | 6.7 | 9.4 | 11.4 | | 13.5 | 11.4 | 8.6 | 9.8 | | 10.7 | 10.2 | 11.1 | 10.2 | | 12.4 | 13.0 | 13.7 | 12.3 | |
| 259 | Lake endemic | Migori | Kuria West | 0.9 | 4.1 | 6.9 | 6.3 | | 5.6 | 5.8 | 4.8 | 1.2 | | 4.4 | 6.4 | 6.0 | 4.1 | | 7.8 | 6.6 | 7.2 | 7.0 | |
| 260 | Lake endemic | Migori | Kuria East | 1.7 | 4.8 | 7.6 | 4.4 | | 3.9 | 4.1 | 2.3 | 3.7 | | 1.6 | 1.0 | 4.2 | 4.3 | | 4.8 | 5.1 | 5.2 | 5.0 | |
| 261 | Highland | Kisii | Bonchari | 1.1 | 5.7 | 7.8 | 9.0 | | 7.6 | 3.7 | 33.4 | 7.6 | | 8.5 | 8.9 | 9.0 | 9.4 | | 11.4 | 10.6 | 11.0 | 10.2 | |
| 262 | Highland | Kisii | South Mugirango | 1.8 | 3.5 | 7.7 | 10.2 | | 9.2 | 9.7 | 9.6 | 8.5 | | 9.2 | 10.7 | 10.7 | 11.0 | | 13.7 | 12.2 | 12.1 | 11.3 | |
| 263 | Highland | Kisii | Bomachoge Borabu | 0.8 | 4.8 | 6.8 | 6.4 | | 4.4 | 4.6 | 4.0 | 4.3 | | 1.2 | 4.1 | 4.9 | 6.3 | | 5.4 | 4.7 | 5.8 | 5.6 | |
| 264 | Highland | Kisii | Bobasi | 0.7 | 3.0 | 3.9 | 5.5 | | 3.8 | 4.2 | 3.4 | 3.0 | | 4.3 | 4.3 | 4.7 | 4.6 | | 5.1 | 4.7 | 5.8 | 9.9 | |
| 265 | Highland | Kisii | Bomachoge Chache | 0.4 | 1.3 | 1.9 | 2.1 | | 11.2 | 2.0 | 1.6 | 5.1 | | 2.0 | 2.3 | 2.1 | 2.3 | | 2.2 | 2.5 | 2.5 | 2.3 | |
| 266 | Highland | Kisii | Nyaribari Masaba | 3.8 | 5.2 | 6.3 | 7.0 | | 6.5 | 6.1 | 5.1 | 6.2 | | 6.7 | 6.9 | 7.1 | 7.5 | | 7.6 | 8.2 | 7.8 | 7.8 | |
| 267 | Highland | Kisii | Nyaribari Chache | 1.6 | 6.8 | 5.2 | 5.5 | | 5.1 | 6.3 | 6.1 | 3.1 | | 5.3 | 5.4 | 5.7 | 6.3 | | 6.0 | 6.7 | 4.3 | 6.7 | |
| 268 | Highland | Kisii | Kitutu Chache North | 2.4 | 14.1 | 19.1 | 22.5 | | 19.4 | 20.8 | 16.8 | 18.8 | | 21.5 | 22.3 | 22.4 | 23.1 | | 23.3 | 25.4 | 25.6 | 24.2 | |
| 269 | Highland | Kisii | Kitutu Chache South | 1.5 | 7.2 | 9.1 | 17.0 | | 24.4 | 10.2 | 8.0 | 9.4 | | 11.1 | 10.7 | 11.4 | 12.3 | | 12.0 | 13.0 | 12.9 | 12.5 | |
| 270 | Highland | Nyamira | Kitutu Masaba | 0.7 | 3.3 | 4.6 | 5.0 | | 4.6 | 4.6 | 3.6 | 3.8 | | 5.3 | 5.0 | 5.2 | 5.3 | | 5.7 | 5.8 | 5.3 | 5.5 | |
| 271 | Highland | Nyamira | West Mugirango | 1.4 | 7.4 | 7.5 | 10.9 | | 11.2 | 10.9 | 7.6 | 14.7 | | 11.0 | 4.2 | 11.8 | 12.3 | | 12.6 | 13.6 | 27.8 | 13.4 | |
| 272 | Highland | Nyamira | North Mugirango | 1.2 | 4.3 | 5.9 | 6.8 | | 6.0 | 6.2 | 4.4 | 5.0 | | 8.2 | 9.0 | 6.8 | 6.5 | | 7.3 | 7.7 | 6.6 | 6.3 | |
| 273 | Highland | Nyamira | Borabu | 0.6 | 2.4 | 5.4 | 3.7 | | 3.3 | 2.6 | 2.6 | 3.0 | | 3.6 | 3.6 | 3.7 | 3.9 | | 3.3 | 4.2 | 3.8 | 3.9 | |
| 274 | Low risk | Nairobi | Westlands | 2.7 | 12.6 | 16.3 | 19.4 | | 17.2 | 17.8 | 15.1 | 43.8 | | 19.2 | 29.2 | 20.6 | 19.9 | | 20.6 | 22.4 | 25.2 | 22.2 | |
| 275 | Low risk | Nairobi | Kilimani | 5.0 | 11.5 | 19.9 | 16.1 | | 11.8 | 2.4 | 13.5 | 4.2 | | 3.2 | 2.7 | 10.3 | 14.6 | | 13.4 | 16.5 | 17.2 | 16.1 | |
| 276 | Low risk | Nairobi | Dagoretti | 5.0 | 15.7 | 21.5 | 24.3 | | 21.5 | 23.4 | 18.6 | 20.0 | | 23.3 | 24.6 | 25.2 | 25.7 | | 26.1 | 28.5 | 28.7 | 27.7 | |
| 277 | Low risk | Nairobi | Langata | 32.2 | 19.7 | 23.8 | 27.4 | | 24.4 | 34.0 | 21.2 | 23.5 | | 25.4 | 25.5 | 27.4 | 18.8 | | 27.7 | 26.0 | 30.1 | 20.3 | |
| 278 | Low risk | Nairobi | Kibra | 2.1 | 13.0 | 12.5 | 13.7 | | 13.0 | 13.1 | 8.7 | 12.4 | | 15.2 | 14.1 | 14.0 | 14.6 | | 14.2 | 16.1 | 16.6 | 15.3 | |
| 279 | Low risk | Nairobi | Roysambu | 2.3 | 9.3 | 11.9 | 13.4 | | 12.0 | 12.6 | 10.4 | 11.3 | | 12.7 | 13.1 | 13.5 | 13.5 | | 14.2 | 12.1 | 13.1 | 14.3 | |
| 280 | Low risk | Nairobi | Kasarani | 3.4 | 15.0 | 20.6 | 23.6 | | 20.9 | 21.9 | 18.7 | 54.2 | | 22.2 | 23.5 | 24.0 | 23.6 | | 25.1 | 27.8 | 28.3 | 25.9 | |
| 281 | Low risk | Nairobi | Ruaraka | 4.0 | 17.5 | 23.5 | 26.1 | | 23.7 | 24.7 | 20.5 | 23.5 | | 26.3 | 27.1 | 28.9 | 30.2 | | 30.0 | 32.4 | 32.3 | 30.4 | |
| 282 | Low risk | Nairobi | Embakasi South | 5.7 | 47.5 | 32.1 | 35.6 | | 32.2 | 35.7 | 27.9 | 30.7 | | 33.6 | 35.7 | 36.2 | 37.8 | | 38.0 | 41.1 | 41.7 | 40.3 | |
| 283 | Low risk | Nairobi | Embakasi North | 1.9 | 10.9 | 21.0 | 21.4 | | 22.3 | 22.4 | 12.1 | 13.8 | | 14.7 | 14.2 | 14.0 | 15.9 | | 17.0 | 19.4 | 16.8 | 17.1 | |
| 284 | Low risk | Nairobi | Embakasi Central | 3.4 | 16.3 | 18.9 | 19.8 | | 17.4 | 19.3 | 18.5 | 18.1 | | 19.8 | 20.8 | 21.1 | 21.5 | | 22.5 | 24.4 | 24.3 | 23.4 | |
| 285 | Low risk | Nairobi | Embakasi East | 10.3 | 27.9 | 37.3 | 41.7 | | 38.0 | 39.8 | 33.9 | 36.0 | | 40.1 | 44.5 | 43.2 | 43.9 | | 44.8 | 48.8 | 49.2 | 42.3 | |
| 286 | Low risk | Nairobi | Embakasi West | 2.7 | 14.5 | 15.4 | 15.9 | | 14.7 | 11.8 | 12.2 | 13.2 | | 14.4 | 15.3 | 16.3 | 16.9 | | 17.0 | 18.8 | 19.1 | 18.0 | |
| 287 | Low risk | Nairobi | Makadara | 2.8 | 13.6 | 19.2 | 20.8 | | 18.4 | 19.5 | 15.9 | 17.9 | | 19.4 | 20.7 | 20.8 | 21.2 | | 21.7 | 23.9 | 24.3 | 23.1 | |
| 288 | Low risk | Nairobi | Kamukunji | 14.6 | 19.2 | 25.8 | 29.2 | | 26.1 | 27.0 | 22.2 | 25.0 | | 28.0 | 28.7 | 29.1 | 56.5 | | 30.7 | 33.7 | 34.4 | 33.0 | |
| 289 | Low risk | Nairobi | Starehe | 13.4 | 10.0 | 13.3 | 15.0 | | 13.3 | 14.2 | 13.5 | 12.4 | | 14.5 | 14.8 | 15.6 | 16.0 | | 15.9 | 17.7 | 17.5 | 16.7 | |
| 290 | Low risk | Nairobi | Mathare | 11.9 | 12.8 | 16.2 | 17.7 | | 16.1 | 17.5 | 17.3 | 15.6 | | 17.6 | 18.4 | 18.5 | 19.1 | | 19.0 | 22.9 | 21.5 | 19.4 | |
